# Pressure‐Induced Coordination Changes in a Pyrolitic Silicate Melt From Ab Initio Molecular Dynamics Simulations

**DOI:** 10.1029/2019JB018238

**Published:** 2019-11-29

**Authors:** N.V. Solomatova, R. Caracas

**Affiliations:** ^1^ CNRS, École Normale Supérieure de Lyon Laboratoire de Géologie de Lyon, CNRS UMR 5276 Lyon France

## Abstract

With ab initio molecular dynamics simulations on a Na‐, Ca‐, Fe‐, Mg‐, and Al‐bearing silicate melt of pyrolite composition, we examine the detailed changes in elemental coordination as a function of pressure and temperature. We consider the average coordination as well as the proportion and distribution of coordination environments at pressures and temperatures encompassing the conditions at which molten silicates may exist in present‐day Earth and those of the Early Earth's magma ocean. At ambient pressure and 2,000 K, we find that the average coordination of cations with respect to oxygen is 4.0 for Si‐O, 4.0 for Al‐O, 3.7 for Fe‐O, 4.6 for Mg‐O, 5.9 for Na‐O, and 6.2 for Ca‐O. Although the coordination for iron with respect to oxygen may be underestimated, the coordination number for all other cations are consistent with experiments. By 15 GPa (2,000 K), the average coordination for Si‐O remains at 4.0 but increases to 4.1 for Al‐O, 4.2 for Fe‐O, 4.9 for Mg‐O, 8.0 for Na‐O, and 6.8 for Ca‐O. The coordination environment for Na‐O remains approximately constant up to core‐mantle boundary conditions (135 GPa and 4000 K) but increases to about 6 for Si‐O, 6.5 for Al‐O, 6.5 for Fe‐O, 8 for Mg‐O, and 9.5 for Ca‐O. We discuss our results in the context of the metal‐silicate partitioning behavior of siderophile elements and the viscosity changes of silicate melts at upper mantle conditions. Our results have implications for melt properties, such as viscosity, transport coefficients, thermal conductivities, and electrical conductivities, and will help interpret experimental results on silicate glasses.

## Key Points

- The proportion and distribution of cation‐to‐oxygen coordination environments in a pyrolite melt have been determined as a function of depth
- We examine the distribution of lifetimes of cation‐oxygen species at select pressure‐temperature conditions
- Our results are compared to previous ab initio calculations on mafic silicate melts and to experiments on various silicate phases

## 1 Introduction

The Early Earth's mantle was once composed of molten silicate rock—a global magma ocean extending down to the core‐mantle boundary (Stevenson, 1989; Tonks & Melosh, 1993). Today, silicate melts exist as lava flows on Earth's surface, as magmas in subterrestial volcanic processes, and are thought to exist at various depths of the mantle, particularly at the base of the upper mantle, at the base of the transition zone, and at the core‐mantle boundary. A seismically detectable low velocity layer observed at the base of the upper mantle is often attributed to the presence of melt within a narrow ~50‐km thick layer (Revenaugh & Sipkin, 1994; Song et al., 2004; Tauzin et al., 2010; Vinnik & Farra, 2007; Freitas et al., 2017). It has also been proposed that dehydration melting occurs at the top of the lower mantle (Schmandt et al., 2014). Silicate melts in the mantle strongly affect seismic velocities, decreasing seismic velocities by several percent (Hammond & Humphreys, 2000). In the deepest part of the lower mantle, at the boundary between Earth's liquid iron‐nickel core and solid D″ layer, it is thought that pockets of silicate melts may exist, explaining some of the seismically observed ultra‐low velocity zones (Vidale & Hedlin, 1998; Williams & Garnero, 1996), though it is not yet clear if they would be gravitationally stable (Bower et al., 2011).

The properties of a melt are strongly dependent on the coordination and geometric packing of its ions. Thus understanding the structure of silicate melts is essential to gain a better understanding of both the Early Earth's magma ocean and for interpreting present‐day experimentally measured properties of amorphous silicates and seismically observed anomalies caused by melts.

Here we conducted simulations at pressures and temperatures relevant to conditions of both the present‐day Earth and the Early Earth's magma ocean. The solidus of dry pyrolite‐like compositions is roughly 1,400–1,500 K at the surface, rising rapidly to 2,000 K by 5 GPa and to 2,500 K by 25 GPa (Hirschmann, 2000; Litasov & Ohtani, 2002), while the solidus of pyrolite at midmantle pressures of 40–80 GPa is about 3,000 K rising to 3,500–4,000 K at core‐mantle boundary pressures (Fiquet et al., 2010; Nomura et al., 2014; Zerr et al., 1998). Thus, our simulation temperatures of 2,000–4,000 K encompass the temperatures at which melt could occur in the entire present‐day mantle. In the Early Earth's magma ocean, the temperature of the molten silicate mantle may have been above the liquidus, which reaches 5,000 K at the base of the lower mantle (Fiquet et al., 2010), and so our calculations at 5,000 K are especially relevant for determining the structure of silicate melts during the early magma ocean stage of Earth.

## 2 Computational Methods

Ab initio molecular dynamics simulations were performed with density functional theory and with the Vienna ab initio simulation package (Kresse & Furthmüller, 1996). We used the projector‐augmented wave method (Blöchl, 1994) to represent the core electrons and the generalized gradient approximation in the Perdew‐Burke‐Ernzerhof form (Perdew et al., 1996) to treat electron exchange and correlation. The electronic structure of iron is calculated at each time step and the d electrons were treated as unconstrained spin polarized at all temperatures and pressures; in other words, the spin state was not enforced to either high or low spin. A Hubbard *U* parameter, which is sensitive to the electronic and geometric environment of iron, was not applied. In previous tests on iron‐bearing pyrolite melt, a *U* value of 4 eV resulted in an increase in the magnetic moment of the iron atoms with only a minor effect on pressure (<1 GPa; Caracas et al., 2019). The kinetic energy cutoffs for the plane‐wave expansion of the wavefunctions and the augmentation charges were set to 550 and 800 eV, respectively. The canonical ensemble with a constant number of atoms, volume, and temperature was used with a Nose‐Hoover thermostat (Nosé, 1984; Hoover, 1985). Simulations were conducted at 2,000, 3,000, 4,000, and 5,000 K and at volumes corresponding to pressures of about 0–40 GPa at 2,000 K and pressures of about 0–160 GPa at 3,000–5,000 K (negative average pressures are achieved when the melt is stretched to a volume slightly beyond the zero‐pressure volume). The Brillouin zone was sampled at the gamma point. Simulations were performed with a time step of 0.5–2 fs for 10–50 picoseconds, depending on the temperature and density. The mean‐square displacement as a function of time shows a ballistic regime below approximately 1,000 fs, after which the atoms reach a diffusive regime.

**Figure 1:**
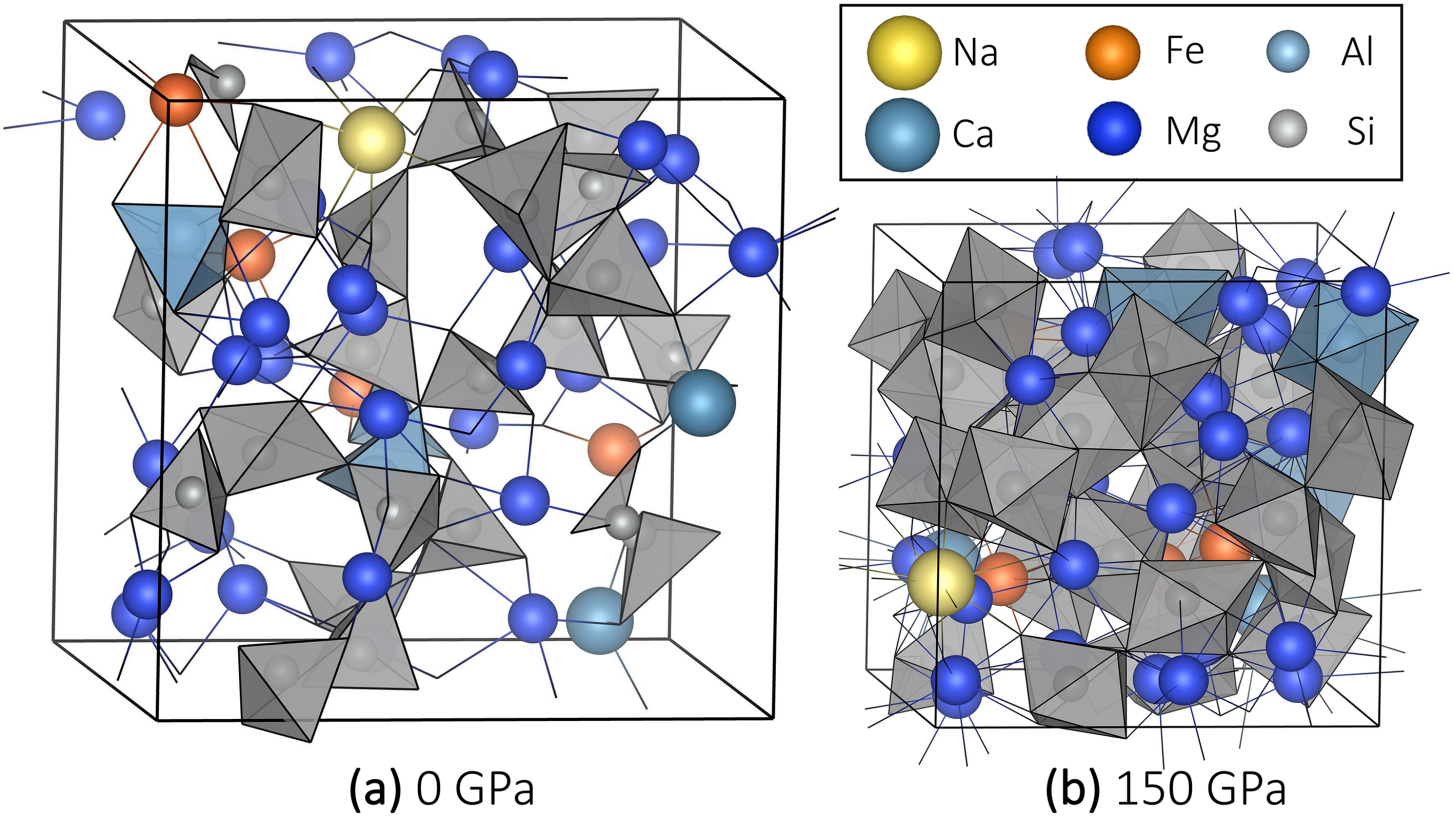
Pyrolite melt at 4,000 K at (a) ~0 GPa and (b) 150 GPa, the highest pressure achieved. SiO_*x*_ and AlO_*x*_ are shown as light gray and light blue polyhedra, respectively, highlighting the transition from predominantly fourfold coordination at low pressure to predominantly sixfold coordination at high pressure.

The composition of the bulk silicate Earth (McDonough & Sun, 1995) was modeled with a pyrolite melt with the stoichiometry NaCa_2_Fe_4_Mg_30_Al_3_Si_24_O_89_ (Figure 1; Table 1). Bond distances were determined from the pair distribution functions (Supporting Information Figure S1), which describe the probability of finding an atom type at a given distance from the reference atom. We use the first peak in the pair distribution function to approximate the average bond length; the distance at which the first minimum occurs marks the radius of the first coordination sphere of atoms that are directly bonded to the reference atom. We use this value to define the bond criterion between two atom types. The maximum acceptable bonds were then fitted with a third‐order polynomial as a function of supercell lattice parameter (Figure S2) to smooth possible statistical irregularities. We chose to fit the maximum bonds as a function of supercell lattice parameter rather than pressure or density due to the even distribution of data points. The fitted values were then used in all speciation analysis. Whether the maximum bonds were fitted or not resulted in a negligible difference in the speciation statistics due to the fact that, by definition, the majority of bonds exist near the peak of the pair distribution function rather than the first minimum.

**Table 1:** Composition of the Pyrolite Melt in Oxide Weight Percent Used for This Study Compared to the Pyrolite Model by McDonough and Sun (1995)

	SiO_2_	Al_2_O_3_	FeO	MgO	CaO	Na_2_O
This study	44.5	4.72	9.00	37.3	3.46	0.96
Pyrolite model	45.0	4.45	8.05	37.8	3.55	0.36

*Note*. The pyrolite model also includes 0.201 wt % TiO_2_, 0.384 wt % Cr_2_O_3_, 0.135 wt % MnO, 0.25 wt % NiO, 0.029 wt % K_2_O, and 0.021 wt % P_2_O_5_, which are not included in our simulations.

## 3 Results

Using ab initio molecular dynamics simulations on pyrolite melt (i.e., a melt with approximately the composition of the bulk silicate Earth) up to core‐mantle boundary pressures and temperatures of 2,000–5000 K, we compute average bond lengths, the distribution of bond lengths, and changes in cation‐oxygen coordination and compare our results to previous experimental and computational studies on amorphous silicates of various compositions.

### 3.1 Bond Lengths

**Figure 2:**
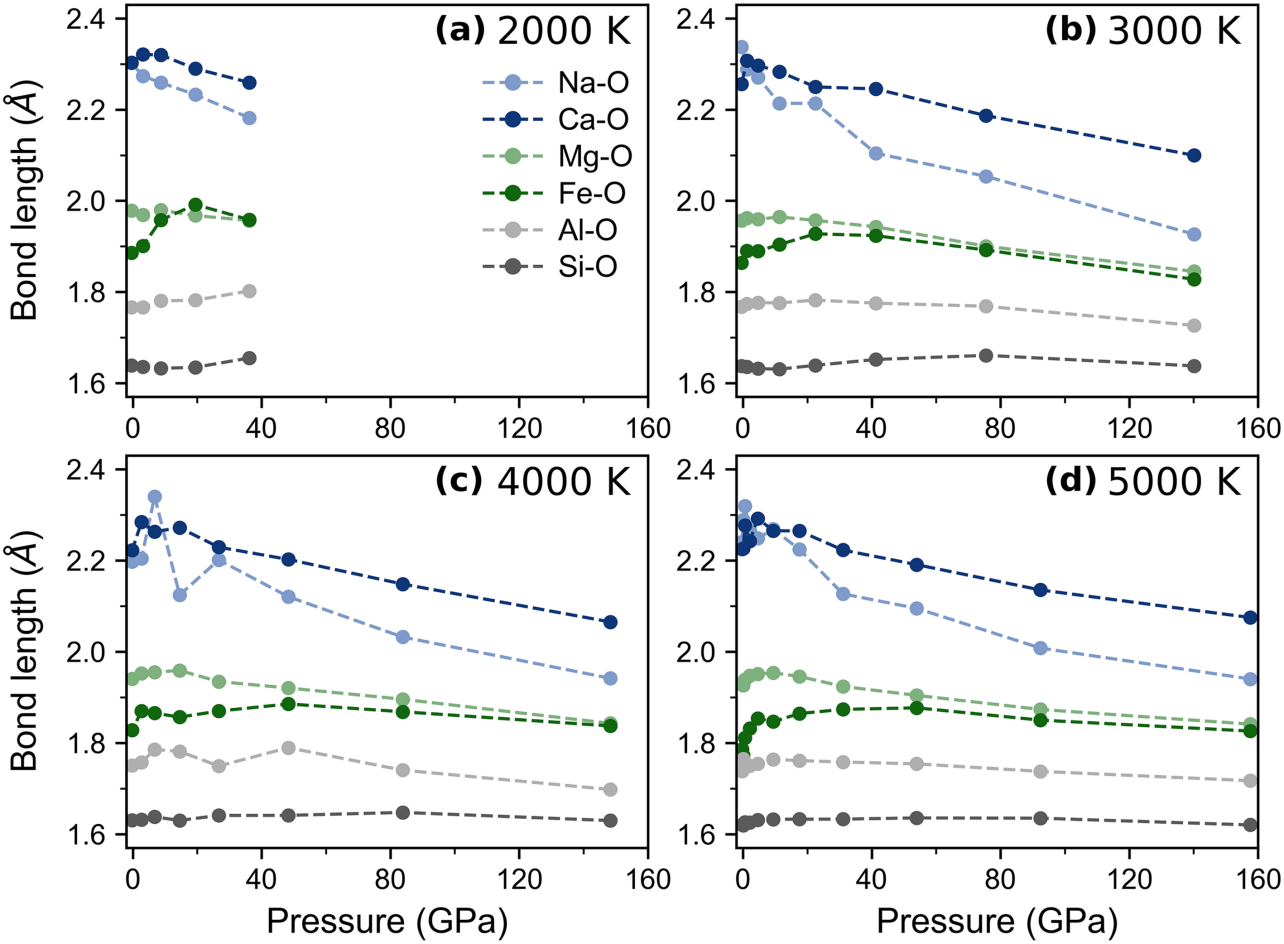
Average bond lengths as a function of pressure, as approximated by the position of the first peak of the corresponding cation‐oxygen pair distribution functions.

The first maximum in the pair distribution function corresponds to the most frequent bond distance that occurs in the simulation and approximately to the average bond length. The maximum acceptable bond distances were determined from the first minimum in the pair distribution function (Figure S2). Using the aforementioned definition, average bond lengths at ambient pressure and 2,000 K are 1.64 Å for Si‐O, 1.77 Å for Al‐O, 1.89 Å for Fe‐O, 1.98 Å for Mg‐O, 2.30 Å for Na‐O, and 2.30 Å for Ca‐O. At upper mantle conditions, the average cation‐oxygen bond lengths for Si‐O, Al‐O, and Mg‐O gently increase with increasing pressure while the average bond lengths for Fe‐O and Ca‐O increase with increasing pressure more steeply (Figure 2). In the same pressure range, the average bond lengths for Na‐O generally decrease with increasing pressure. At transition‐zone and lower‐mantle pressures (i.e., above roughly 15 GPa), the bond lengths for all cation‐oxygen pairs decrease monotonically with increasing pressure (Figure 2). The slope of the change in bond distance with depth is dependent on a balance between the increase in pressure and increase in coordination. At low pressures, bond distances increase slightly with increasing pressure because the coordination number and polyhedra size increase very rapidly (section 3.2) while at high pressures, bond distances decrease with increasing pressure as the ions get closer together and the polyhedra shrink in size.

### 3.2 Atomic Coordinations

The average coordination of all cations with respect to oxygen generally increases with increasing pressure. At upper mantle conditions, calcium and sodium undergo sharp increases in coordination while silicon and aluminum undergo more gentle increases in coordination and at lower mantle pressures, the coordination environments of calcium and sodium remain roughly constant. The average coordination of all cations with respect to oxygen at all pressures and temperatures, as well as the errors associated with pressure are provided in Tables S1–S4.

#### 3.2.1 Silicon‐Oxygen

**Figure 3:**
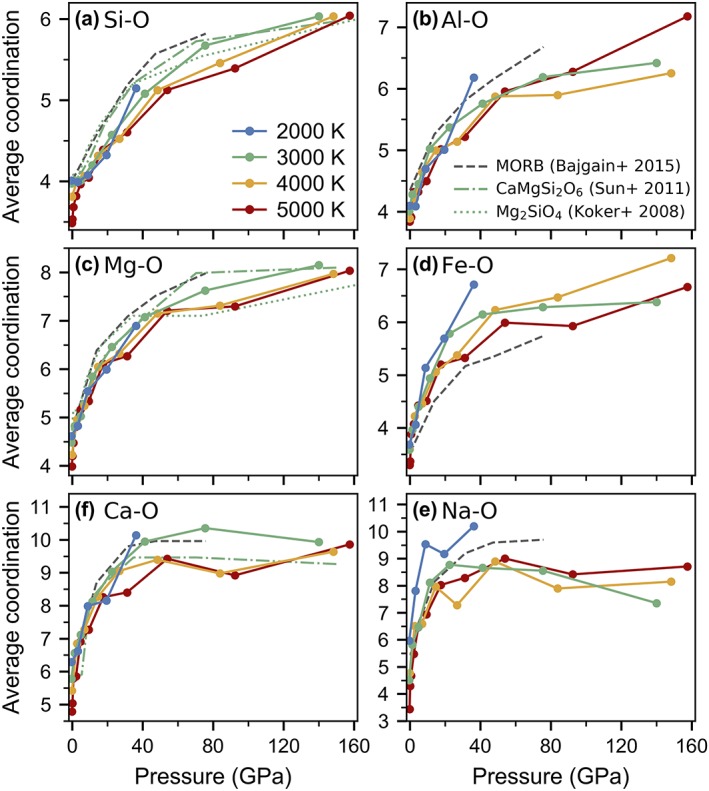
Average coordination numbers of cations with respect to oxygen as a function of pressure for cation‐oxygen pairs: (a) Si‐O, (b) Al‐O, (c) Mg‐O, (d) Fe‐O, (e) Ca‐O, and (f) Na‐O. Solid lines with solid symbols are the results of this study where blue is 2,000 K, green is 3,000 K, yellow‐orange is 4,000 K and red is 5,000 K. Ab initio molecular dynamics simulations on midocean ridge basalt (MORB) melt are shown as a gray dashed line, as reported averaged over temperatures of 1,800–4,000 K and pressures at each volume (Bajgain et al., 2015). Ab initio molecular dynamics simulations at 3,000 K on CaMgSi_2_O_6_ and Mg_2_SiO_4_ melts are shown in green dashed‐dotted and dotted curves, respectively (Sun et al., 2011; de Koker et al., 2008). The average coordination of silicon from ab initio calculations on MgSiO_3_ melt at 3,000 K (Stixrude & Karki, 2005) overlaps nearly perfectly with the silicon's average coordination in CaMgSi_2_O_6_ (Sun et al., 2011) and so is not shown here for clarity.

**Figure 4:**
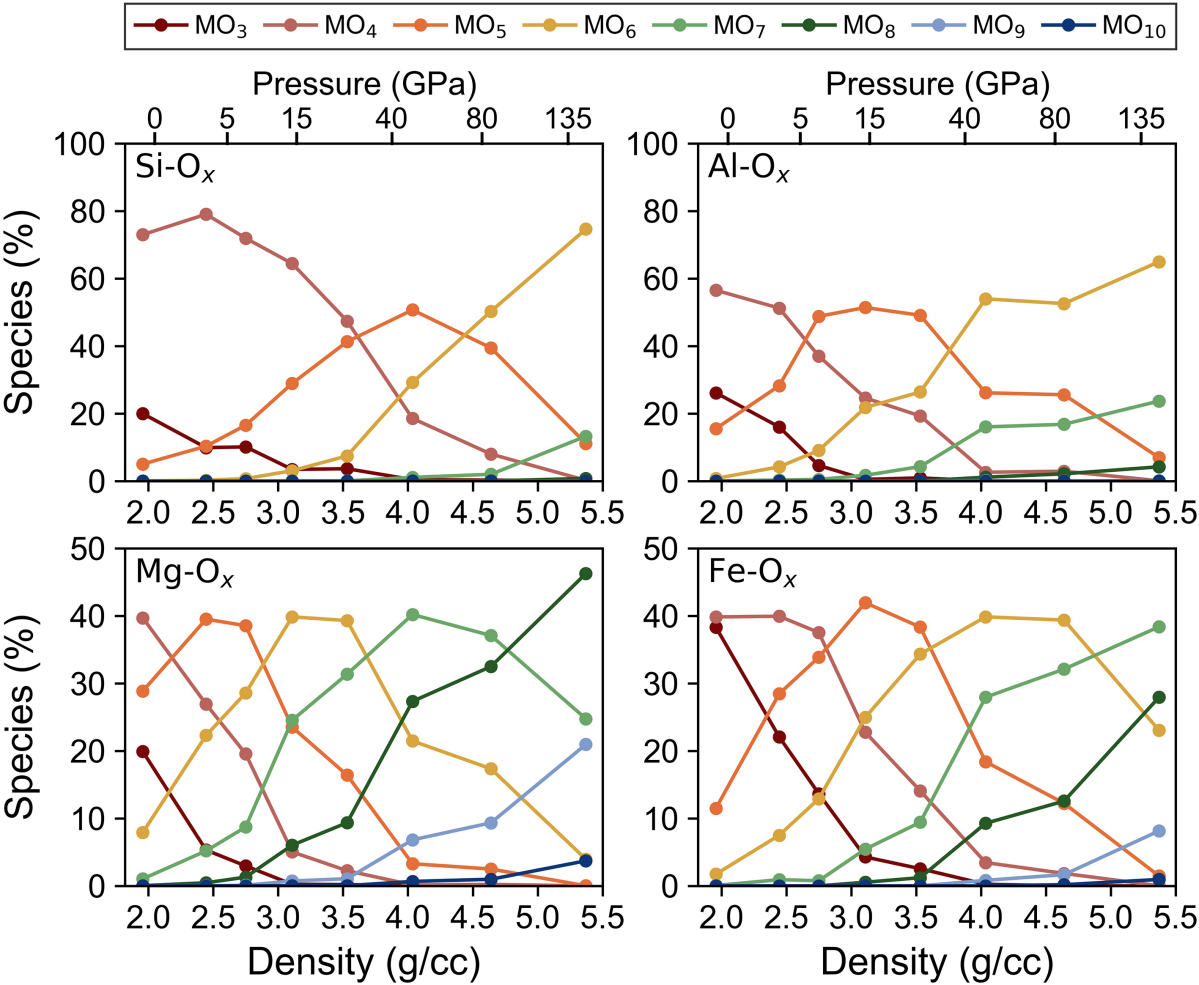
Atomic coordination of (a) silicon, (b) aluminum, (c) magnesium, (d) and iron to oxygen at 4,000 K as a function of density (corresponding pressures are provided on top). Coordination numbers are labeled next to each curve. See Supporting Information Figure S4 for the atomic coordination of silicon, aluminum, and magnesium at 2,000 K and 3,000 K compared to 4,000 K.

**Figure 5:**
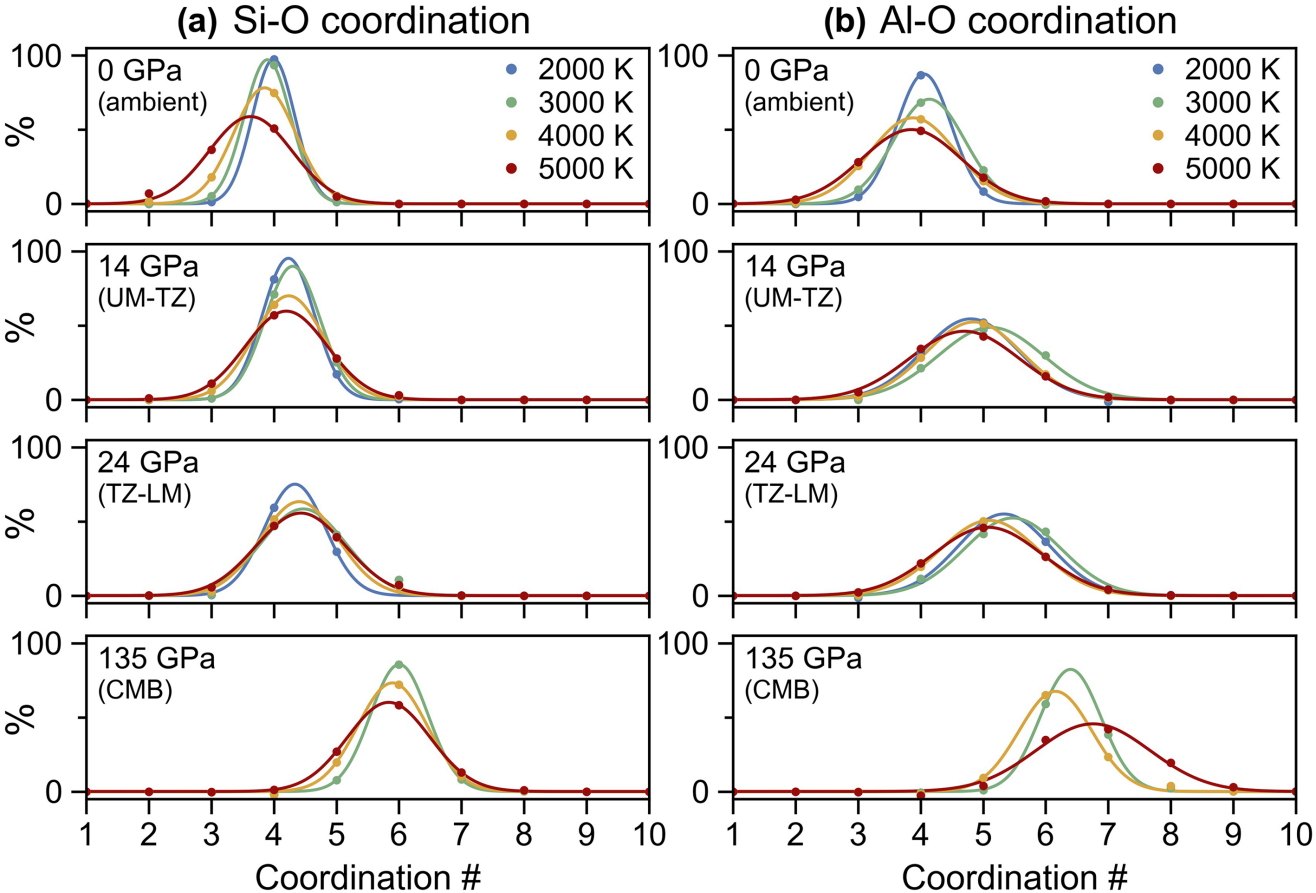
The distribution of coordination environments for (a) silicon and (b) aluminum with respect to oxygen at pressures of 0 GPa (ambient pressure), 14 GPa (base of the upper mantle, UM), 24 GPa (base of the transition zone, TZ), and 135 GPa (core‐mantle boundary, CMB) and temperatures of 2,000–5,000 K. Calculations were performed at constant volume and temperature (see computational methods in section 2 for more details); to determine the coordination numbers at exactly the same pressures for all temperatures, the coordination numbers were locally fitted along isotherms as a function of pressure with a third‐order polynomial.

As the most abundant cation in silicate melts, the chemical environment of silicon is most strongly linked to the properties of the melt, such as density and viscosity, and it is therefore essential to understand changes in coordination of silicon as a function of depth. At ambient pressure and 2,000 K, we find that silicon has an average coordination of 4, existing almost entirely as SiO_4_ tetrahedra (Figure 3a). With increasing temperature and constant ambient pressure, the average coordination approaches 3 as the proportion of fourfold‐coordinated silicon decreases at the expense of threefold‐coordinated silicon. The proportions of the most common coordination environments of silicon with respect to oxygen are plotted in Figure 4a at 4,000 K and at all temperatures in Figures S3 and S4. Only about 50% of silicon has fourfold coordination at 5,000 K compared to nearly 100% at 2,000 K. Unlike at 2,000 K, where the average coordination of silicon remains constant at pressures of 0–5 GPa, at 5,000 K, the average coordination increases rapidly from threefold to fourfold within the first 5 GPa. Overall, with increasing temperature, the distribution of coordination environments increases while the average coordination decreases (Figure 5a). Fitting a Gaussian curve to the coordination numbers at 2,000 K produces a full width at half maximum (FWHM) of 0.8, ranging from 3.6 to 4.4, whereas at 5,000 K, the full width at half maximum increases to 1.5, ranging from 2.9 to 4.4.

With increasing pressure, the average coordination number of silicon increases with a general trend from fourfold at low pressure to fivefold at 40–50 GPa to sixfold at core‐mantle boundary pressures. At 5,000 K, the proportion of fourfold‐coordinated silicon increases with increasing pressure up until 5 GPa, after which, it steadily decreases. Meanwhile, the proportion of threefoldcoordinated silicon decreases with pressure, reaching 0% by about 50 GPa, and the proportions of fivefold‐coordinated silicon steeply increases with pressure. Sixfold‐coordinated silicon remains under 1% abundance until 10 GPa and under 5% abundance until 20 GPa for all temperatures, after which, its abundance increases rapidly. At core‐mantle boundary pressures, the proportion of sixfold silicon is about 80% at 3,000 K, 70% at 4,000 K, and 55% at 5,000 K.

#### 3.2.2 Aluminum‐Oxygen

At ambient pressure, despite having a larger average bond length compared to silicon, aluminum displays a similar coordination environment to silicon, existing predominantly in fourfold coordination at 2,000 K and decreasing slightly in coordination and increasing in distribution of coordination environments at higher temperatures. However, with increasing pressure, the proportion of fourfold‐coordinated aluminum decreases more rapidly than the proportion of fourfold‐coordinated silicon (Figure 3b). By 40–50 GPa, there is no fourfold‐coordinated aluminum left in the melt, whereas a small fraction of fourfold‐coordinated silicon persists up to lowermost mantle pressures. The proportion of fivefold‐coordinated aluminum increases with increasing pressure reaching a maximum of about 50% at 10–20 GPa (depending on the temperature), after which, the population of fivefold‐coordinated aluminum decreases to 5% by core‐mantle‐boundary pressures (Figure 4b). The proportions of sixfold and sevenfold aluminum increase with increasing pressure. Between 50 and 100 GPa, roughly 50% of aluminum is sixfold coordinated, 25% is fivefold coordinated, and 25% is sevenfold coordinated, where differences in temperature appear to be mostly a result of scatter due to the small quantity of aluminum atoms in the melt. The proportions of the most common coordination environments for aluminum with respect to oxygen are plotted as a function of pressure in Figure S4, while the distribution of the coordination environments at select pressures are plotted in Figure 5b.

#### 3.2.3 Magnesium‐Oxygen

**Figure 6:**
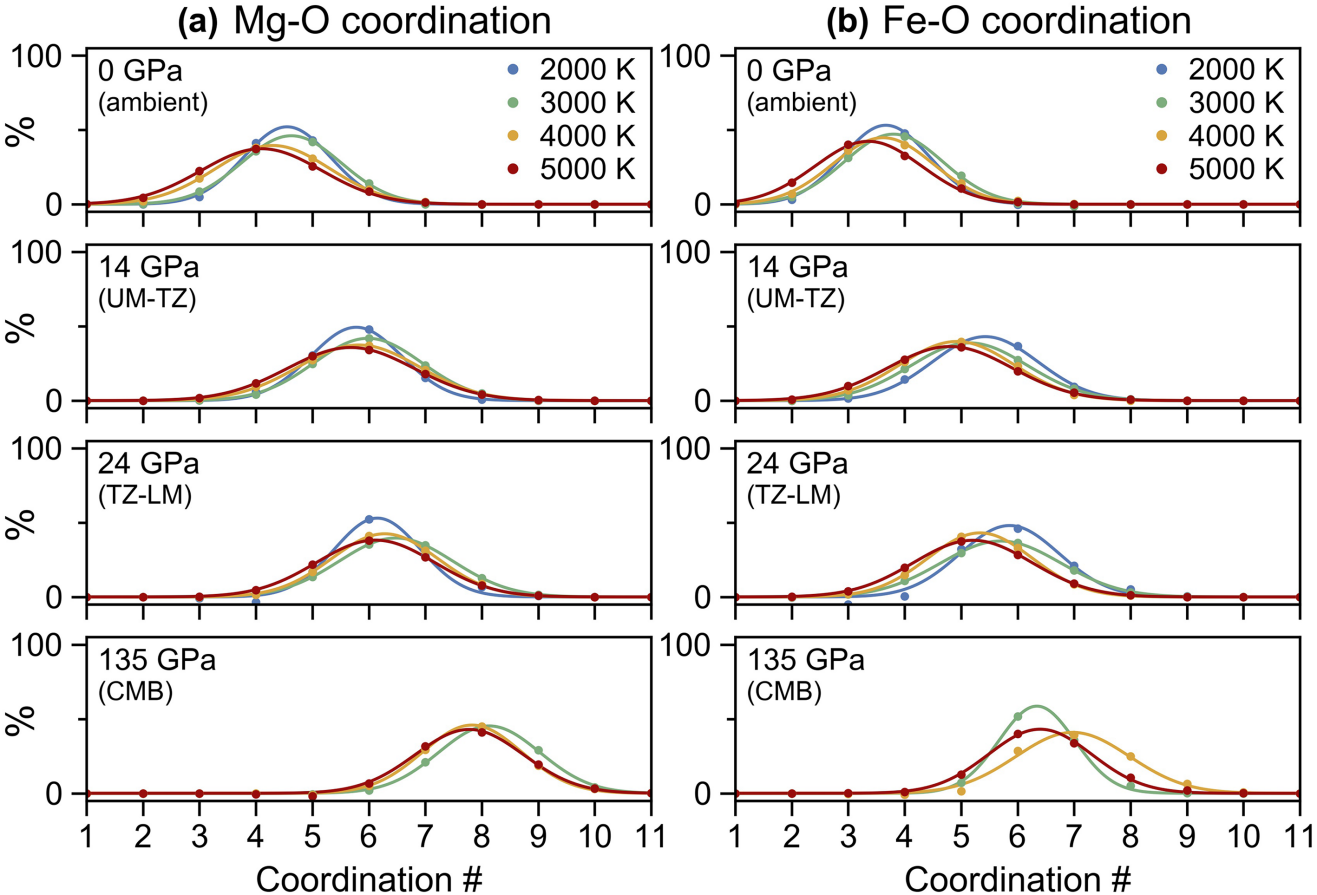
The distribution of coordination environments for (a) magnesium (b) and iron with respect to oxygen at pressures of 0 GPa (ambient pressure), 14 GPa (base of the upper mantle, UM), 24 GPa (base of the transition zone, TZ), and 135 GPa (core‐mantle boundary, CMB) and temperatures of 2,000–5,000 K. Calculations were performed at constant volume and temperature (see computational methods in section 2 for more details); to determine the coordination numbers at exactly the same pressures for all temperatures, the coordination numbers were locally fitted along isotherms as a function of pressure with a third‐order polynomial.

We find that at ambient pressure and 2,000 K, the average coordination of magnesium in pyrolite melt is 4.6 (Figure 3c), existing as a mixture of threefold (10%), fourfold (40%), fivefold (40%), and sixfold (10%) coordination (Figures S4 and S6). With increasing pressure, the average coordination of magnesium increases to sixfold by 20 GPa, sevenfold by 45 GPa, and nearly eightfold by 135 GPa. There is generally a negative correlation between temperature and average coordination number. At ambient pressure, average coordination of magnesium decreases from 4.6 at 2,000 K to 4 at 5,000 K while at 40 GPa, it decreases from about 7 at 2,000 K down to 6.6 at 5,000 K (Figure 3c). The distribution of environments is also slightly and positively correlated with temperature; interestingly though, at core‐mantle boundary pressures, temperature no longer affects the distribution (Figure 6a). The proportion of fourfold‐coordinated magnesium decreases rapidly at low pressure to under 5% by 20 GPa, while the concentrations of fivefold, sixfold, and sevenfold coordination increase with pressure and reach a maximum at about 3, 20, and 50 GPa, respectively, after which, their concentrations decrease with increasing pressure as higher coordination begin to dominate (Figures 4c and S6).

#### 3.2.4 Iron‐Oxygen

Although it might be expected that the average coordination of iron would be higher than the average coordination of magnesium, ab initio molecular dynamics simulations of silicate melts seem to predict a lower average coordination (Bajgain et al., 2015; this study). Non‐spin‐polarized calculations on midocean ridge basalt (MORB) melt produced an average iron to oxygen coordination of 3.5 at 0 GPa increasing to 4.5 by about 14 GPa (Bajgain et al., 2015). In comparison, in our spin‐polarized calculations on pyrolite melt, the average coordination of iron at 2,000 K increases from 3.7 at 0 GPa to 5.5 at 15 GPa; by 25 GPa, iron is on average sixfold coordinated (Figure 3d). At core‐mantle boundary conditions, iron is approximately sixfold to sevenfold coordinated. The proportions of the most common coordination environments for iron with respect to oxygen are plotted as a function of pressure in Figure S7, and the distribution of the coordination environments at select pressures are plotted in Figure 5b. At ambient pressure, iron has a magnetic moment of about 3.5 μB where the iron atoms remain entirely in the high‐spin state. With increasing pressure, the magnetic moment of iron gradually decreases. An example of the distribution of local magnetic moments of iron as a function of pressure is shown in Figure S8.

#### 3.2.5 Calcium‐Oxygen

**Figure 7:**
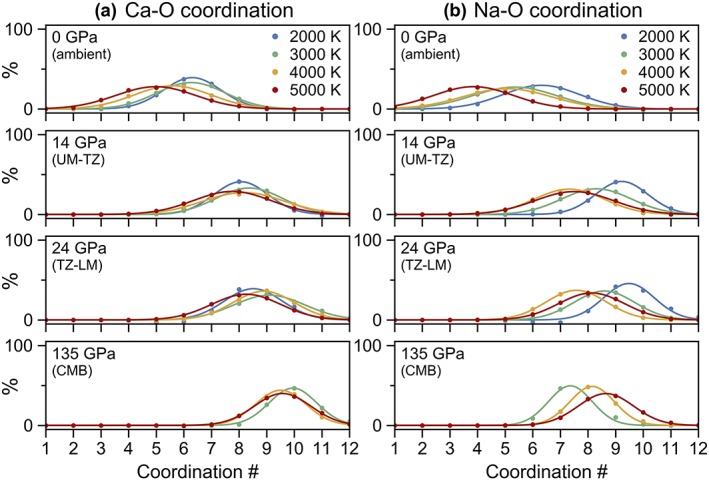
The distribution of coordination environments for (a) calcium (b) and sodium with respect to oxygen at pressures of 0 GPa (ambient pressure), 14 GPa (base of the upper mantle, UM), 24 GPa (base of the transition zone, TZ), and 135 GPa (core‐mantle boundary, CMB) and temperatures of 2,000–5,000 K. Calculations were performed at constant volume and temperature (see computational methods in section 2 for more details); to determine the coordination numbers at exactly the same pressures for all temperatures, the coordination numbers were locally fitted along isotherms as a function of pressure with a third‐order polynomial.

Having a much larger ionic radius than magnesium or iron, calcium exhibits a higher coordination environment, which also increases in coordination more rapidly with pressure (Figure 3f). At ambient pressure, calcium exhibits a very broad distribution of coordination environments, ranging from fourfold to eightfold at 2,000 K and threefold to sevenfold at 5,000 K (Figure 7a). At the base of the upper mantle, 20% of calcium is sevenfold, 40% is eightfold, 25% is ninefold, and 10% is tenfold (the remaining 5% is fivefold and elevenfold). With increasing pressure, the distribution of coordination environments narrows. For example, at core‐mantle boundary pressure, calcium exists mostly in ninefold (30%) and tenfold (45%) coordinations with lesser proportions of eightfold (10%) and elevenfold (15%) coordinations (Figure S9). At 2,000 K, the average coordination of calcium by oxygen increases steeply from 6.3 to 8 with increasing pressure from 0 to 10 GPa while with increasing temperature, the average coordination drops from 6.3 to about 5 at 2,000 to 5,000 K (Figure 3f). The average coordination of calcium increases to 7 by 6 GPa and 8 by 15 GPa, independent of temperature. Above 40 GPa, the average coordination number flattens out at around 9–10 up to core‐mantle boundary conditions.

#### 3.2.6 Sodium‐Oxygen

Due to the low concentration of sodium (one atom per 153‐atom supercell), the statistics for sodium are poor and the scatter in the data is subsequently large, so results should be evaluated cautiously. In general, sodium behaves similarly to calcium. At low pressure, the average coordination ranges from 6 at 2,000 K down to 3.5 at 5,000 K. The average coordination of sodium increases rapidly to 8–9 by 15 GPa, after which, the average coordination plateaus at around 8 (Figure 3e). Similar to calcium, the distribution of coordination environments is very large at low pressure and sharpens at high pressure as the coordination of sodium by oxygen atoms is maximized (Figure 7b). The proportions of the most common coordination environments for sodium with respect to oxygen are plotted as a function of pressure in Figure S10.

#### 3.2.7 Lifetimes of Species

**Figure 8:**
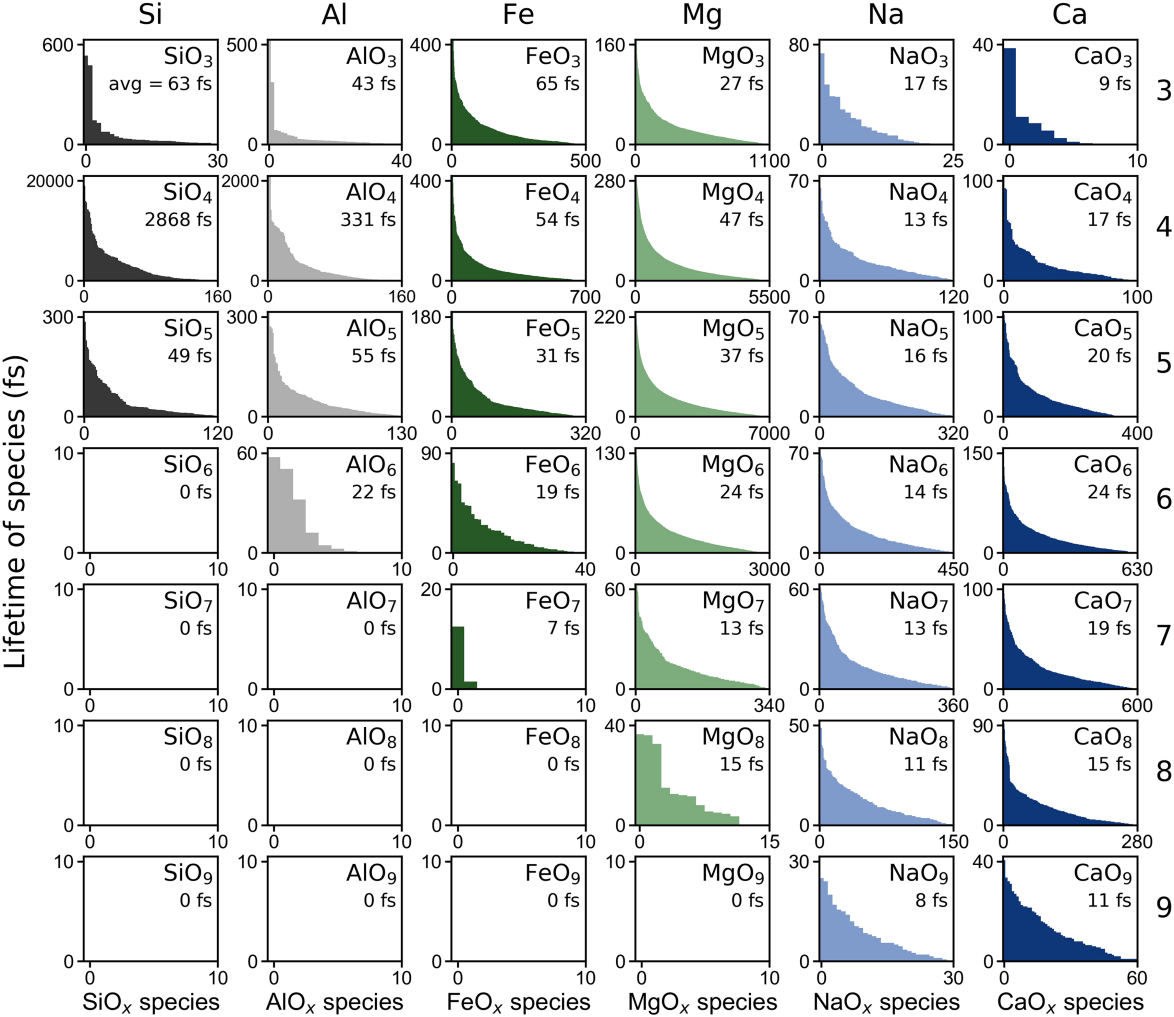
Lifetimes of each species at ambient pressure (~0 GPa) and 2,000 K. The *x* axis represents each individual MO_*x*_ species sorted in decreasing order of lifetimes, and the *y* axis is the lifetime (the amount of time each species lived for) in femtoseconds. The average length of time that each type of species existed for in femtoseconds is labeled within each figure. The total simulation length was 20,600 fs. For example, within the simulation, there were about 160 SiO_4_ species that existed for 0–20,600 fs with an average lifetime of about 2,900 fs. In contrast, there were 5,500 MgO_4_ species that existed for only 0–280 fs with average lifetime of about 50 fs.

**Figure 9:**
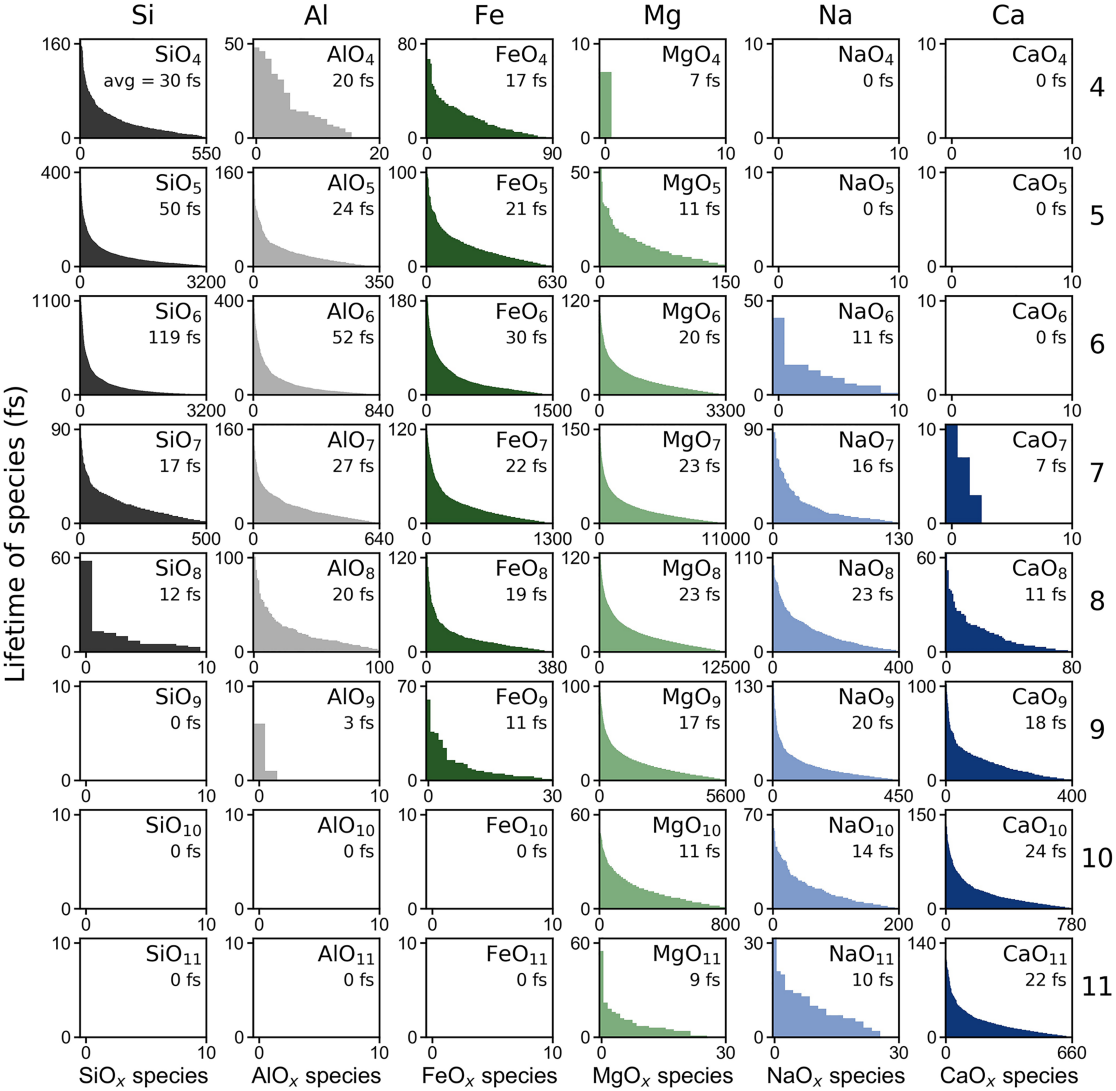
Lifetimes of each species at 80 GPa and 3,000 K. The *x* axis represents each individual MO_*x*_ species sorted in decreasing order of lifetimes, and the *y* axis is the lifetime (the amount of time each species lived for) in femtoseconds. The average length of time that each type of species existed for in femtoseconds is labeled within each figure. The total simulation length was 23.9 picoseconds.

In a molecular dynamics simulation, bonds are formed and broken as the ions move through the melt, forming various types of species. Here we focus on cation‐anion species, MO_*x*_ (where M is one of the six cations, O is oxygen, and *x* is the number of oxygen atoms surrounding the cation, as defined by the first coordination shell in the pair distribution function). Each MO_*x*_ species lives for some amount of time before the coordination changes, and we refer to the time they exist for as the “lifetime” of the species. Some species, such as SiO_4_ tend to live for very long times (thousands of femtoseconds), while others are much more short lived (tens of femtoseconds). We performed a careful analysis of the lifetime of MO_*x*_ species. In Figure 8, we show the lifetimes of species at ambient pressure (~0 GPa) and 2,000 K. The longest surviving SiO_4_ species survives the entirety of the simulation (20,646 fs) with an average SiO_4_ lifetime of 2,868 fs. Although aluminum has a similar coordination environment to silicon at these conditions, AlO_4_ tetrahedra are not trapped within as deep of a potential minimum as SiO_4_, and so the longest surviving AlO_4_ species only lives for about a fifth of the simulation time lives (4.805 fs) and has an average lifetime of 331 fs. Behaving more like network modifiers even in low coordination environments, iron and magnesium polyhedra have much shorter lifetimes, ranging from 20 to 65 fs for the more populous threefold to sixfold coordinations while sodium and calcium have even shorter lifetimes of 10–25 fs for the threefold to eightfold coordinations. Na‐O and Ca‐O bonds break and reform frequently as the relatively weakly bonded oxygen atoms migrate through the melt much more easily than the oxygen atoms bonded to silicon tetrahedra. As temperature increases, the lifetimes of species decreases. For example, at 0 GPa and 4,000 K, the average lifetime of SiO_4_ tetrahedra is 244 fs, more than an order of magnitude lower than at 2,000 K (Figure S11). At midmantle solidus conditions, many different types of coordination environments exist for silicon with respect to oxygen. At 80 GPa and 3,000 K, for example, silica tetrahedra exist for an average of 30 fs while silica octahedra exist for an average of 119 fs (Figure 9), decreasing down to 16 and 43 fs, respectively, at 150 GPa and 5,000 K (Figure S12). Aluminum‐oxygen polyhedra, ranging from fourfold to eightfold coordination exist on average for about 20–50 fs at 80 GPa and 3,000 K, decreasing down to 15–20 fs at 150 GPa and 5,000 K. Iron‐oxygen and magnesium‐oxygen polyhedra with fivefold to eightfold coordination exist on average for 10–30 fs and similarly, sodium‐oxygen and calcium‐oxygen polyhedra with eightfold to elevenfold coordination exist on average for 10–25 fs at 80 GPa and 3,000 K (Figure 9), decreasing further by 150 GPa and 5,000 K (Figure S10).

## 4 Discussion

We compare our results to ab initio molecular dynamics simulations on a MORB melt (Bajgain et al., 2015), Mg_2_SiO_4_ melt (de Koker et al., 2008), CaMgSi_2_O_6_ melt (Sun et al., 2011), and MgSiO_3_ melt (Stixrude & Karki, 2005). We note that Bajgain et al. (2015) averaged their coordination numbers over pressures corresponding to each volume and temperatures of 1,800–4,000 K (resulting in an average temperature of ~3,000 K). For Mg_2_SiO_4_ (de Koker et al., 2008) and CaMgSi_2_O_6_ (Sun et al., 2011), we compare results at 3,000 K. The average cation‐anion coordinations are systematically higher in MORB melt (Bajgain et al., 2015) compared to pyrolite melt for all cations except for iron, which has a lower coordination number at all pressures, likely due to the difference in spin. Our simulations are spin polarized (section 2 for more details), whereas the MORB simulations are non‐spin polarized, resulting in a smaller ionic radius and consequently, smaller coordination. It has been shown in previous calculations on pyrolite that iron remains in a magnetized state for most of the pressures and temperatures of the magma ocean (Caracas et al., 2019).

We also compare our results to experiments on silicate glasses. The coordination environment of cations with respect to oxygen has been determined experimentally using a wide range of techniques, including infrared spectroscopy, nuclear magnetic resonance spectroscopy (NMR), small‐angle X‐ray scattering, and inelastic X‐ray Raman scattering.

### 4.1 Coordination Environments

#### 4.1.1 Silicon‐Oxygen

In crystalline materials, silicon is generally fourfold coordinated by oxygen at upper mantle pressures (e.g., forsterite and enstatite) (Smith, 1959; Smyth & Hazen, 1973) and increases to sixfold in the lower mantle (e.g., periclase and bridgmanite) (Hazen, 1976; Ito & Matsui, 1978). Unlike crystalline materials, melts have a distribution of coordination environments, which smoothly increase with increasing pressure. Silicon exists in mostly fourfold coordination at ambient pressure and temperatures of 2,000–4,000 K in pyrolite melt (Figure 3a), in agreement with ab initio simulations on MORB (Bajgain et al., 2015; Karki et al., 2018), Mg_2_SiO_4_ (de Koker et al., 2008), CaMgSi_2_O_6_ (Sun et al., 2011), and MgSiO_3_ melts (Stixrude & Karki, 2005). At 2,000 K and ambient pressure, 1% of silicon in pyrolite melt exists in threefold coordination, 1% exists in fivefold coordination, and the remaining 98% exists in fourfold coordination. At higher temperatures, the amount of threefold‐coordinated silicon increases, which has never been experimentally investigated, mostly due to the fact that experiments are limited in temperature. Although Karki et al. (2018) do not explicitly report threefold silicon in MORB melt, at 3,000 K and ambient pressure, it is reported that 91% of silicon is in fourfold coordination and 5% of silicon is in fivefold coordination with an average coordination of 4, indicating that 4% of silicon is in threefold coordination. At equivalent conditions, we predict that 5% of silicon is threefold, 93% is fourfold, and 1% is fivefold, in agreement with the results of Karki et al. (2018) despite the different melt chemistries. The nontrivial amount of threefold‐coordinated silicon at >3,000 K suggests that it is more relevant than previously thought, particularly in the Early Earth's magma ocean where the surface temperatures were much above the liquidus, especially if an atmosphere was forming.

With compression at upper mantle conditions, the coordination of silicon in pyrolite melt increases at a slower pace than of other cations, remaining close to fourfold over a large pressure range due to the deep potential minimum of silica tetrahedra. We find that with further increasing depth, its average coordination number increases following a general trend from fourfold at shallow depths (<10 GPa and 2,000 K) to fivefold at midmantle depths (40–50 GPa and 3,000 K) to sixfold at core‐mantle boundary conditions (135 GPa and 4,000 K). At core‐mantle boundary pressures, the proportion of sixfold silicon decreases with increasing temperature from 80% at 3,000 K to 70% at 4,000 K to 55% at 5,000 K. The proportions of silicon in fourfold, fivefold, and sixfold coordination in pyrolite melt are essentially identical to the proportions in CaMgSi_2_O_6_ melt at all pressures (Figure S3). Compared to simulations on MORB melt (Bajgain et al., 2015; Karki et al., 2018), the proportions of fourfold and fivefold silicon are slightly larger in pyrolite melt and CaMgSi_2_O_6_ melt (Sun et al., 2011) while the proportion of sixfold silicon is a bit lower because of the different degrees of polymerization or M: Si ratios (where M is Mg, Ca, Fe, and other network‐modifier cations), which cumulatively explains the larger average coordination of silicon in MORB melt. The average coordination of silicon at high pressure is likely dependent on the degree of polymerization of the melt. At ambient pressure, the ratio, nonbridging oxygen per tetrahedrally coordinated cation (NBO/T) is often used to quantify the degree of polymerization such that olivine is completely depolymerized with an NBO/T ratio of 4, pyrolite is moderately depolymerized with an NBO/T ratio of 2.6, MgSiO_3_ has an NBO/T ratio of 2, MORB is relatively polymerized with an NBO/T ratio of about 0.7, and SiO_2_ and feldspars are fully polymerized with an NBO/T ratio of 0. Although at high pressure the NBO/T ratio is no longer valid, the relative polymerization of the melts at ambient pressure gives insight to their relative behavior at high pressure.

Using the first peak of the radial distribution function for MgSiO_3_ glass, which corresponds to the combined influence of the coordination environments of silicon and magnesium with respect to oxygen, Kono et al. (2018) suggest that the coordination of silicon increases from fourfold to sixfold to about 70 GPa, after which, it remains constant at sixfold up to about 110 GPa. In our simulations on pyrolite melt, the coordination of silicon increases to 5.6 by 75 GPa and up to 6 by 140 GPa at 3,000 K without a plateau, although the density of data is not high enough to eliminate the possibility of a plateau between data points. Sanloup, Drewitt, Crépisson, et al. (2013) conducted X‐ray diffraction experiments on basalt melt up to 60 GPa, finding that the coordination of silicon increases to sixfold by 35 GPa and plateauing at an average coordination number of 6.2 up to 60 GPa. In contrast, silicon is still fivefold coordinated in our simulations on pyrolite melt at 35 GPa and 2,000–3,000 K, likely due to the lower degree of polymerization in pyrolite melt.

Using mid‐infrared absorption spectroscopy on fully polymerized glasses (silica [SiO_2_], anorthite [CaAl_2_Si_2_O_8_], and diopside [CaMgSi_2_O_6_]), Williams and Jeanloz (1988) observed a gradual increase in the coordination of silicon from entirely fourfold at 0 GPa to increasingly sixfold at higher pressures. At 40 GPa, the highest pressure achieved, the silica and diopside glasses contained coordination environments ranging from fourfold to sixfold, whereas the anorthite glass appeared to contain no fourfold silicon by 40 GPa. With inelastic X‐ray Raman scattering, Lin et al. (2007) demonstrated that silicon in SiO_2_ glass undergoes a coordination change from fourfold like to sixfold like between 10 and 22 GPa; however, the spectra for the glass are much broader than the spectra for the crystalline quartz and stishovite standards, which is explained by the possible presence of fivefold coordination. The combined effect of broadening and increasing of the coordination environments may also yield the same effect. Compared to these results on silica glass, the fourfold to sixfold transition in silicon in pyrolite melt occurs at much higher pressures.

Several studies have calculated the average coordination of silicon with respect to oxygen in SiO_2_ glass using X‐ray diffraction, generally finding that the coordination number of silicon increases from fourfold to sixfold by about 40–50 GPa, followed by a more gradual increase in coordination (Maede et al., 1992; Sato & Funamori, 2010; Brazhkin et al., 2011; Prescher et al., 2017); however, slight differences in the trends were observed. Using average Si‐O distances in SiO_2_ glass measured with X‐ray diffraction and comparing to crystalline materials, Meade et al. (1992) found that silicon exists almost entirely in fourfold coordination between 0 and 10 GPa, increasing rapidly towards sixfold between 10 and 40 GPa. Prescher et al. (2017) also find that the coordination of silicon in SiO_2_ plateaus at fourfold between 0 to 14 GPa, after which, it increases steeply to sixfold by about 50 GPa. Similarly, in our simulated pyrolite melt at 2,000 K, we observe a plateau at fourfold coordination between 0 and 10 GPa, after which, it rises sharply to fivefold by 40 GPa, the highest pressure achieved. In contrast, Brazhkin et al. (2011) observe a steep continuous increase in coordination from 4 to 5.7 between 0 and 40 GPa without a plateau in both the compression and decompression regimes. Above 50 GPa, Brazhkin et al. and Prescher et al. observe a shallow increase in coordination up to about 6.75 by core‐mantle boundary pressures while Sato and Funamori (2010) observe a plateau in coordination at sixfold up to about 100 GPa. Although in our simulations, we also observe a change of slope at around 50 GPa, the overall coordination numbers at 3,000–5,000 K are lower, reaching a maximum of sixfold coordination by core‐mantle boundary pressures. The difference is likely due to the different chemistries (e.g., the presence of network‐modifying cations in pyrolite melt and thus much lower degree of polymerization) and to a lesser extent, the higher temperatures in our simulations.

While the pressure‐induced coordination change of silicon by oxygen from fourfold to sixfold is well documented in both silicate melts and silicate minerals (Liu & Bassett, 1986), fivefold silicon rarely occurs in crystalline phases and has consequently received less attention in silicate melts. Fivefold silicon is rare even in high‐pressure crystalline phases, with the most notable example in CaSi_2_O_5_ (Kanzaki et al., 1991; Angel, 1997); instead, silicon in minerals typically shows either fourfold or sixfold coordination or a mixture of the two (Finger & Hazen, 2000). In minerals, the pressure‐induced progression from fourfold to sixfold is either direct or occurs via a mixed‐coordination phase. For example, silicon was observed to transition from entirely fourfold to mixed fourfold and sixfold to entirely sixfold coordination in forsterite as it transforms to metastable high‐pressure polymorphs (Finkelstein et al., 2014). Unlike crystalline materials, melts and glasses have a continuous distribution of coordination environments, which gradually increase in coordination with increasing pressure, and thus include coordinations that are rarely observed in minerals, such as fivefold and sevenfold coordinations.

Even at ambient pressure, a small proportion of fivefold‐coordinated silicon was experimentally observed in various alkali silicate glasses, with the proportion of fivefold‐coordinated silicon increasing with increasing pressure and temperature. For example, NMR experiments on K_2_Si_4_O_9_ and Na_2_Si_4_O_9_ glasses found that 0–0.16% of silicon was fivefold in glasses quenched from ambient pressure and 3–4% of silicon was found to be fivefold in glasses quenched from 6 GPa (Stebbins & McMillan, 1993) while NMR experiments on sodium aluminosilicate glasses observe that 0.3–3% of silicon is fivefold in ambient‐pressure glasses and 0–3% is sixfold in glasses synthesized at 6 GPa (Kelsey et al., 2009). Consistent with the experiments, we predict that at 2,000 K, about 1% of Si is fivefold at 0 GPa and 4% is fivefold at 6 GPa (from extrapolation between the data points at 3 and 9 GPa). Our simulations show that the proportion of fivefold‐coordinated silicon continues to increase with a maximum proportion of fivefold coordination at about 40–50 GPa, accounting for nearly 50% of SiO_*x*_ species at all temperatures (Figure 5a).

#### 4.1.2 Aluminum‐Oxygen

Aluminum typically substitutes for tetrahedrally coordinated silicon in silicate crystals (e.g., in feldspars) but may behave as both a network former and a network modifier in silicate melts, even at ambient pressure. We find that at ambient pressure, aluminum displays a similar coordination environment to silicon, existing predominantly in fourfold coordination at ambient pressure, but displays a slightly broader distribution environment. For example, at 2,000 K and ambient pressure, 5%, 87%, and 8% of aluminum exists in threefold, fourfold, and fivefold coordinations, respectively, compared to 1%, 98%, and 1% for silicon, respectively. At higher temperatures, the amount of threefold and fivefold aluminum increases to 10% and 23%, respectively. Although Karki et al. (2018) do not report threefold aluminum in MORB melt, at 3,000 K and ambient pressure, it is reported that 66% of aluminum is in fourfold coordination and 24% of aluminum is in fivefold coordination with an average coordination of 4.2, indicating that 8% of aluminum is in threefold coordination, in agreement with our predictions despite the different chemistries.

Compared to silicon, the coordination of aluminum increases much more rapidly with depth. By 500 km, the average coordination is fivefold and by 1,200 km, it is sixfold with no fourfold aluminum remaining (in contrast to silicon, which exists in fourfold coordination even at lowermost mantle pressures, albeit in small proportions). A similar trend was observed in ab initio simulations of MORB melt (Bajgain et al., 2015; Karki et al., 2018). Compared to pyrolite melt, the proportions of sixfold and sevenfold aluminum in MORB melt increase slightly more rapidly with pressure at the expense of fourfold and fivefold aluminum, resulting in an overall larger average coordination, likely as a result of a larger alumina concentration compared to pyrolite (16 wt % compared to 4.7 wt % Al_2_O_3_).

Experiments on aluminosilicate and tectosilicate glasses have demonstrated that the coordination of aluminum is mostly fourfold with the proportion of fivefold‐coordinated aluminum ranging from 0% to 30%, depending strongly on composition, but generally increasing with increasing alumina content (Risbud et al., 1987, Sato et al., 1991; Poe et al., 1992; Toplis et al., 2000; Sen & Youngman, 2004; Neuville, Cormier, Flank, et al., 2004; Neuville, Cormier, Massiot, 2004; Neuville et al., 2006; Thompson & Stebbins, 2011; Malfait et al., 2012) and with increasing content of modifying cations (Allwardt et al., 2005; Bunker et al., 1991; Kelsey et al., 2009; Morin et al., 2014; Poe et al., 1993). Kelsey et al. (2009) conducted a detailed NMR study on a series of sodium aluminosilicate glasses synthesized at 1 atm and 6 GPa. In all the glasses synthesized at 1 atm, fivefold‐coordinated aluminum was present without any sixfold‐coordinated aluminum whereas in the glasses synthesized at 6 GPa, both fivefold and sixfold aluminum were observed. The average coordination of aluminum was found to be fourfold for all glasses at 0 GPa but ranged from 4.6 to 5.5 at 6 GPa. We predict that at 2,000 K, aluminum's average coordination is 4 at 0 GPa and increases to about 4.4 by 6 GPa where 55% is fourfold, 35% is fivefold, and 5% is sixfold. In comparison, Kelsey et al. observed proportions of 58% fourfold, 28% fivefold, and 14% sixfold in the glass with the lowest average aluminum coordination of 4.6, and Bajgain et al. (2005) predicted roughly 45% fourfold, 35% fivefold, and 15% sixfold in MORB melt at 6 GPa from extrapolation.

Several studies have observed a minimum in the magnitude of the viscosity and/or plateauing of the viscosity between 0.5 and 6 GPa (Kushiro 1986; Poe et al., 1997; Suzuki et al., 2005; Allwardt et al., 2007). It has been hypothesized that the viscosity minimum may correspond to the maximum abundance of fivefold aluminum (Poe et al., 1997; Poe & Rubie, 2000). However, we observe a maximum in fivefold aluminum at 10–20 GPa (where the maximum increases with increasing temperature; Figures S4 and S5). The viscosity minimum may instead be related to the stagnation of the formation of SiO_6_ (Figure S3), which in our study remains at ~0% abundance between 0 and 9 GPa, after which, it increases steeply to 36% abundance by 36 GPa.

#### 4.1.3 Magnesium‐Oxygen

Despite the different melt compositions, our prediction that the average coordination of magnesium by oxygen increases from about 4.5 at ambient pressure to nearly 8 by 135 GPa is in agreement with ab initio simulations on MORB melt (Bajgain et al., 2015), Mg_2_SiO_4_ melt (de Koker et al., 2008), and CaMgSi_2_O_6_ melt (Sun et al., 2011; Figure 3c). In fact, at 3,000 K and pressures of about 40–120 GPa, the average coordination of magnesium in pyrolite melt generally falls between the average coordination of magnesium in Mg_2_SiO_4_ and CaMgSi_2_O_6_ melts such that there is a general decrease in magnesium's average coordination with increasing magnesium content at approximately lower mantle pressures.

X‐ray scattering, NMR, and Mg K‐edge X‐ray absorption near edge structure (XANES) experiments have found that the average coordination of magnesium in ambient‐pressure silicate glasses varies from fourfold to sixfold, depending on the chemistry (Shimoda et al., 2008; Trcera et al., 2009). The average coordination of magnesium was found to be 4.1–4.5 in MgSiO_3_ enstatite glass (Waseda & Toguri, 1977; Yin et al., 1983), 4.8 in Mg_3_Al_2_Si_3_O_12_ pyrope glass (Okuno & Marumo, 1993), and 5 in diopside and albite glasses (Ildefonse et al., 1995; Dien et al., 1999). As a magnesium‐rich glass, the calculated average coordination of 4.6 in pyrolite melt is consistent with the experimental studies on the enstatite and pyrope glass systems.

In crystalline phases, magnesium has a much larger coordination number. At low pressure, magnesium has a coordination number of 6 in pyroxenes (Warren & Bragg, 1929; Smith, 1959) and 8 in garnets (Novak & Gibbs, 1971) while in bridgmanite of the lower mantle, magnesium exists in a mixture of octahedral and highly distorted dodecahedral sites (Dobson & Jacobsen, 2004; Jephcoat et al., 1999), yet we find that in mafic silicate melts, the average coordination of magnesium remains below eightfold in most of the lower mantle, indicating that the packing of silicate melts is less efficient than crystalline materials. Even though the coordination of silicon in melts is similar to silicon in crystalline phases and the coordination increases at a comparable rate, network‐modifying cations are persistently surrounded by fewer oxygen atoms than in crystalline phases at equivalent pressure‐temperature conditions.

#### 4.1.4 Iron‐Oxygen

In ambient‐pressure minerals, ferrous iron is typically in sixfold coordination (Smyth & Hazen, 1973; Cameron et al., 1973) while in silicate melts and glasses, ferrous iron exists in fourfold, fivefold, and sixfold coordinations, depending on composition as well as interpretation of experimental results. Using radial distribution functions from high‐temperature X‐ray diffraction experiments, Waseda et al. (1980) calculated coordination numbers of iron ranging from 4.3 to 5.7 in FeO‐Fe_2_O_3_‐SiO_2_ melts (increasing with decreasing silica content) and with Mössbauer spectroscopy and Fe K‐edge XANES on a wide variety of ferrosilicate glasses, Jackson et al. (2005) found that the average coordination of ferrous iron is as low as 3.9 in sodium‐iron silicate glass and as high as 5.2 in basaltic glass. With similar results, a detailed Mössbauer spectroscopy study on aluminosilicate glasses suggests that ferrous iron is mostly fourfold in sodium aluminosilicate glasses and fivefold to sixfold in calcium and magnesium aluminosilicate glasses (Mysen, 2006). Ferrous iron was also found to be in fourfold coordination in Na_2_FeSi_3_O_8_ and K_2_FeSi_3_O_8_ glasses (Waychunas et al., 1988), Fe_0.4_Mg_0.8_Ca_0.8_Si_2_O_6_ glass (Calas & Petiau, 1983), and Fe_2_SiO_4_ glass (Cooney & Sharma, 1990) with X‐ray absorption spectroscopy; however, in a careful X‐ray diffraction study on fayalite melts, Sanloup, Drewitt, Konôpková, et al. (2013) found that ferrous iron has an average coordination number of 4.8 at 0 GPa and 7.2 at 7.5 GPa.

With XANES and multiple‐scattering simulations on submarine basaltic glass, Wu et al. (1999) observed iron in predominantly fourfold coordination with lesser amounts of fivefold‐coordinated iron and no indication of sixfold coordination. Rossano et al. (2000) found that ferrous iron exists in fourfold and fivefold coordinations in CaFeSi_2_O_6_ glass using a combination of extended X‐ray absorption fine structure and molecular dynamics simulations. Comparing Na‐, K‐, and Al‐bearing melts to glasses with X‐ray absorption spectroscopy, Wilke et al. (2007) found an average coordination close to 5, with a slightly higher concentration of fourfold coordinated iron in melts with respect to glasses. Iron was interpreted to be predominantly in sixfold coordination in albite‐diopside glasses (Keppler, 1992), basalt glass (Bell & Mao, 1974), and a variety of Fe‐Ti glasses (Nolet et al., 1979) with optical absorption spectroscopy. Using time‐domain Mössbauer and optical absorption spectroscopy on reduced basaltic glass, Solomatova et al. (2017) found that less than 15–20% of ferrous iron is in fourfold coordination and the rest of iron is consistent with fivefold and/or sixfold coordination. Thus, although the proportions of the site environments depend on composition, the reported coordination environments also depend strongly on the experimental technique and method of analysis and interpretation.

Ab initio calculations on silicate melts have found that iron has an average coordination of about 3.5–3.7 at ambient pressure and 2,000‐3,000 K (Bajgain et al., 2015; this study), below experimentally determined values. Two main possibilities may explain the discrepancy: the coordination of iron in glasses is lower than in melts (e.g., Wilke et al., 2007) or density functional theory fails to accurately predict the geometric environment of iron likely due to its failure to accurately treat the d electrons. Indeed, it is difficult to imagine that iron has a lower coordination compared to magnesium, especially considering that the difference is not trivial (a difference of one oxygen atom at ambient pressure in our simulations).

#### 4.1.5 Calcium‐Oxygen

XANES and X‐ray diffraction experiments on calcium‐bearing silicate glasses have found that at ambient pressure, calcium bonds to oxygen with a wide range of bond distances, ranging from 2.29 to 2.85 Å with an average coordination of about 7, ranging from 6 to 7.4, depending on the experimental technique and composition of the glass (Taylor & Brown, 1979; Okuno & Marumo, 1993; Eckersley et al., 1988; Cormier et al., 2000; Cormier & Neuville, 2004, Neuville, Cormier, Massiot, 2004). However, the proportions of calcium coordination environments in amorphous silicates have not yet been determined experimentally. Our results demonstrate that calcium reaches an average coordination of 9–10 by roughly 40 GPa and stays roughly constant up to core‐mantle boundary conditions, whereas calcium in majorite garnet is already twelvefold within the upper mantle (Hazen et al., 1994).

#### 4.1.6 Sodium‐Oxygen

At ambient pressure and temperature of 2,000 K, the coordination of sodium is 6 but increases very rapidly with pressure. By the base of the upper mantle, it reaches a maximum of 8–9. The coordination of sodium in crystalline low albite is fivefold or sevenfold (Phillips et al., 1988), bounding our determined coordination of sixfold in ambient‐pressure pyrolite. In amorphous silicates, experimentally determining sodium‐oxygen bond distances can be arbitrary as the first and second coordination spheres are continuous rather than distinct. NMR experiments on sodium‐rich silicate glasses and melts have estimated that the average coordination of sodium is about 6–8 without a dependence on composition (Maekawa et al., 1997; Xue & Stebbins, 1993).

#### 4.1.7 Partitioning of Minor Elements Between the Molten Iron and Silicate Melt

The structure of silicate melts likely affects the partitioning behavior of minor elements between silicate melt and iron metal (Hillgren et al., 1996; Keppler & Rubie, 1993; Mann et al., 2012). The core‐to‐mantle partition coefficient of highly and moderately siderophile elements decreases with increasing pressure more steeply between 0 and ~6 GPa and more shallowly above ~6 GPa (Kegler et al., 2008; Mann et al., 2012). In pyrolite melt, we observe several sharp changes in the atomic coordination environments within the first 10 GPa. Up to about 10 GPa, the concentration of sixfold‐coordinated silicon remains below 1%, after which, its concentration increases steeply. At 2,000 K, the concentration of sixfold‐coordinated silicon reaches 4% at 20 GPa and 36% at 36 GPa while at 3,000 K, the concentration of sixfold‐coordinated silicon is 7% at 23 GPa and 41% at 27 GPa (Figure S3). Meanwhile, the concentrations of fivefold‐coordinated magnesium and sevenfold‐coordinated calcium reach a local maximum such that their concentrations increase with increasing pressure up to 3–5 GPa at 2,000–3,000 K, accounting for roughly 45% and 35% of their corresponding species, and decrease with increasing pressure up to 40 GPa, at which point, their relative concentrations reach 0% (Figures S6 and S9). These coordination environments may provide a favorable environment for siderophile elements within the silicate melt; and so, the steep increase in their abundance at low pressure may be responsible for the steep slope observed in their core‐to‐mantle partition coefficients below ~6 GPa. Furthermore, experiments on carbon‐bearing iron melt have shown a compressibility increase at about 5 GPa (Sanloup et al., 2011). Thus, we believe that the combined structural changes in silicate and iron melt may be responsible for the kink in the partitioning behavior between the two melts (Keppler & Rubie, 1993; Mann et al., 2012).

## 5 Conclusions

Using ab initio molecular dynamics calculations, we performed a detailed analysis of the local coordination environments of the major cations in pyrolite melt, a good approximation of the bulk silicate Earth composition (McDonough & Sun, 1995). We considered the pressure and temperature conditions spanning the entire Earth's mantle as well as the conditions of the magma ocean that were prevalent during the Early Earth, comparing our results to previous experimental and computational studies on amorphous silicates.

We find a broad distribution of the cation‐oxygen coordination environments. The sharpest distribution is found in silicon at uppermost mantle conditions, occupying predominantly fourfold tetrahedral coordination while the broadest distribution of site environments is found in calcium and sodium at upper mantle conditions, sharpening slightly at higher pressures as the coordination is effectively maximized. The average coordination numbers for the cations with respect to oxygen generally increase with increasing pressure more steeply in the upper mantle and more gradually in the lower mantle. We computed the lifetimes of all the species present in our simulations. At 2,000 K and ambient‐pressure conditions, we find SiO_4_ tetrahedra as the longest‐living species, with an average close to 3 picoseconds while AlO_4_ tetrahedra exist for an average of 0.3 picoseconds. In contrast, the lifetimes of most of the coordination polyhedra of the network‐modifying cations, that is Fe, Mg, Ca, and Na, are on the order of a few tens of femtoseconds at most. As temperature increases, the lifetimes of the species decrease, as more the atoms have more kinetic energy, and bonds are more easily broken and reformed.

We observe a non‐negligible contribution from exotic coordination environments (relative to crystalline Earth materials), such as threefold‐coordinated cations at ambient pressure and temperatures of 3,000 to 5,000 K, predominantly fivefold‐coordinated aluminum at midmantle conditions, and significant amounts of sevenfold coordination of all cations at lowermost mantle conditions. In fact, this study is the first computational study to highlight the presence of threefold‐coordinated cations, which likely played a significant role in the physical properties of the Early Earth's magma ocean. Sharp changes in coordination behavior of fivefold‐coordinated magnesium and sevenfold‐coordinated calcium at about 3–5 GPa and the sharp increase in sixfold‐coordinated silicon above 10 GPa may explain a kink found in experiments in the pressure dependence of the partitioning coefficient of minor elements between the magma ocean and liquid iron. Our detailed structural results may also be used to improve geodynamic modeling of planetary magma oceans, as the density, viscosity, and compressibility behavior of melts are directly linked to the geometric packing of its ions.

## Supporting information

Supporting Information S1

Data Set S1
