# Supplementary material for: Pressure‐Induced Coordination Changes in a Pyrolitic Silicate Melt From Ab Initio Molecular Dynamics Simulations
Source: J Geophys Res Solid Earth. 2019 Nov 29;124(11):11232–50. doi: 10.1029/2019JB018238 (PMC6988478; doi:10.1029/2019JB018238)
Supplement: Supplementary file 1 — Supporting Information S1 [file JGRB-124-11232-s001.pdf]

AGU PUBLICATIONS

Journal of Geophysical Research: Solid Earth

Supporting Information for

Pressure-induced coordination changes in a pyrolitic silicate melt from ab initio molecular dynamics simulations

N.V. Solomatova[1] and R. Caracas[1]

[1]CNRS, École Normale Supérieure de Lyon, Laboratoire de Géologie de Lyon, UMR 5276, Lyon, France.

Contents of this file

Figures S1 to S12
Tables S1 to S4

Introduction

Here we provide the pair distribution functions for all cation-oxygen pairs at ambient pressure and 2000-5000 K (Fig. S1). Pair distribution functions were used to determine the maximum cation-oxygen distances, plotted in Fig. S2 for the speciation analysis. We show the detailed proportions of all cation-oxygen species as a function of pressure (Figs. S3-S7, S9-S10) and we plot two additional examples of the lifetimes of all cation-oxygen species at 0 GPa & 4000 K and at 150 GPa & 5000 K (Figs. S11-S12). In Fig. S8 we show the distribution of local magnetic moments in iron as a function of pressure and in Tables S1-S4 we list the average coordination of all species as plotted in Fig. 3 of the main text.

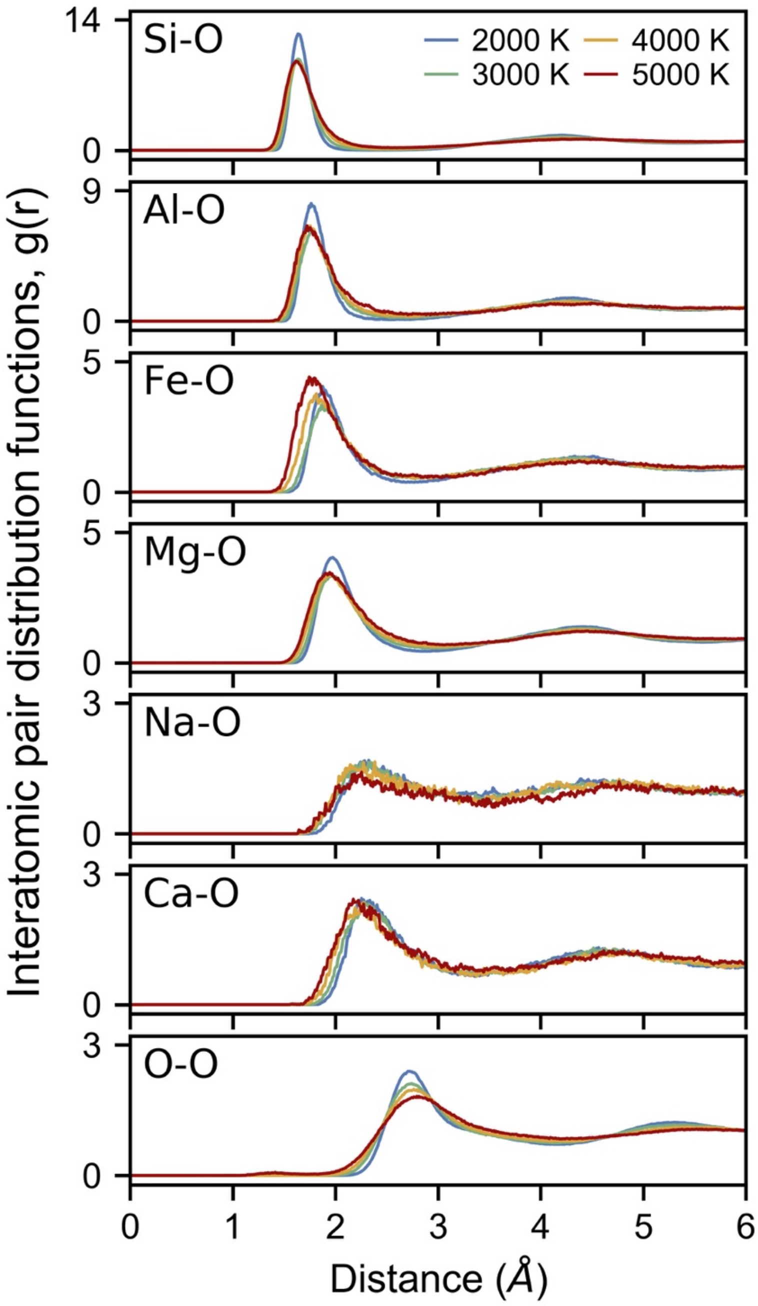

Figure S1. Interatomic pair distribution functions (g(r)) for ion-oxygen pairs at approximately ambient pressure and temperatures of 2000-5000 K.

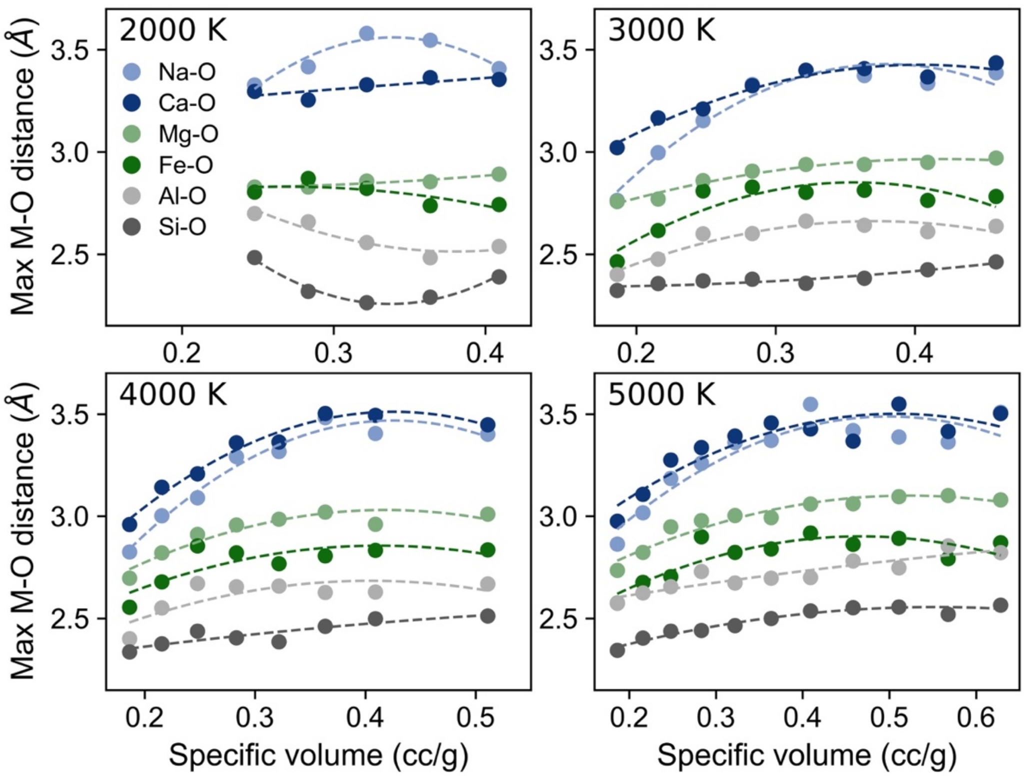

Figure S2. Maximum cation-oxygen (M-O) distances as a function of specific volume fitted with a third-order polynomial. The fitted values were used in the speciation analysis to remove scatter arising from differences in statistical sampling of the simulations at various densities.

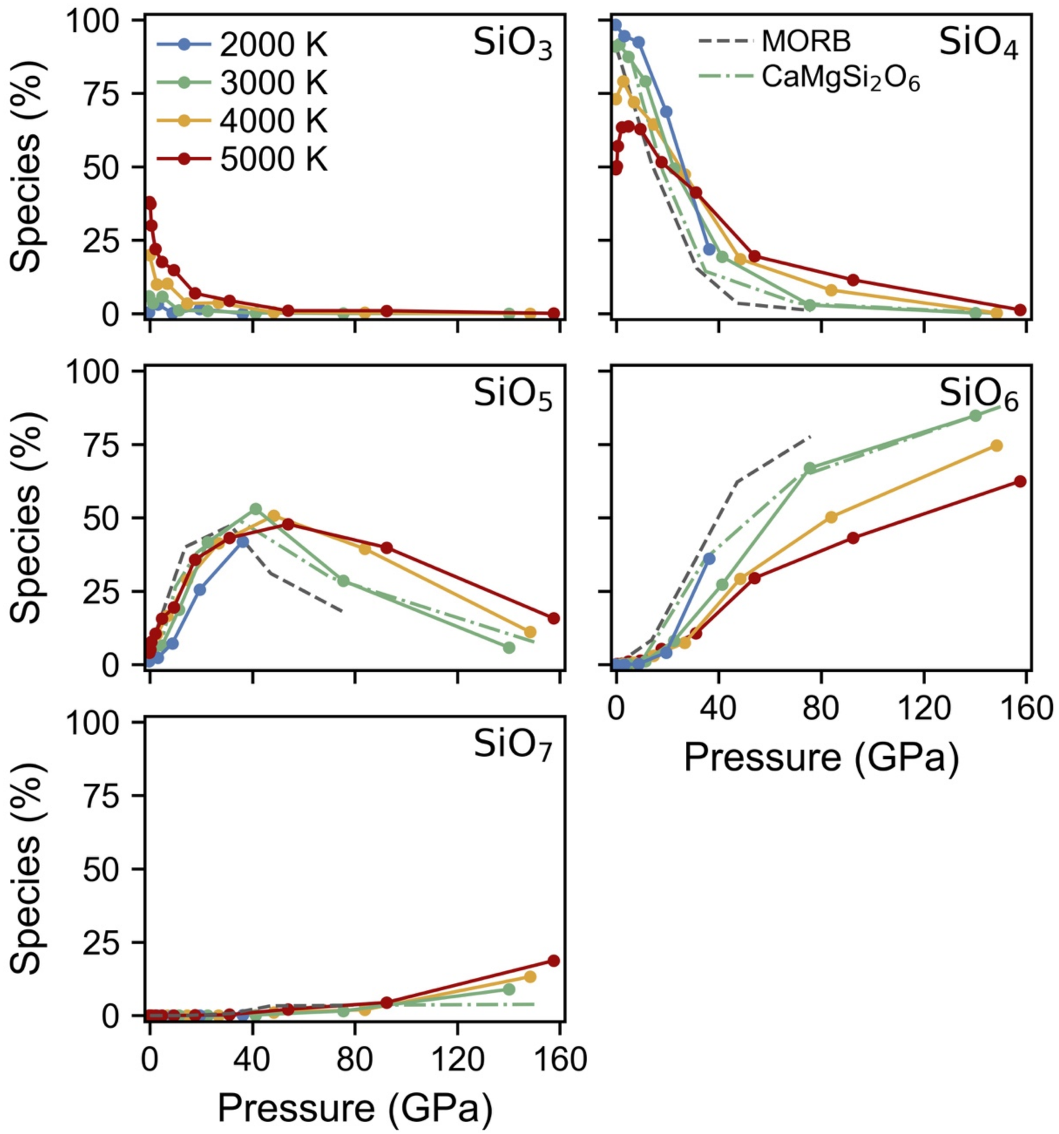

Figure S3. The proportions of the five most common species of SiO_x as a function of pressure. It is important to note that coordination numbers are not distinct in a melt; instead they exist as a continuous distribution of coordination environments. Pair distribution functions were used to determine cutoffs for the bond-lengths (see Computational Methods for more details) and the above data points were then used to create Fig. 6, which illustrates the continuity of the coordination numbers. The proportions of SiO_x species from ab initio molecular dynamics calculations on MORB melt (Bajgain et al., 2015) and $CaMgSi_2O_6$ (Sun et al., 2011) are shown as the dashed gray curve and dashed-dotted green curve, respectively. MORB data was averaged over temperatures of 1800-4000 K and pressures at each volume (Bajgain et al., 2015). The coordination proportions for silicon in $MgSiO_3$ melt (Stixrude and Karki, 2005) overlap with those in $CaMgSi_2O_6$ melt (Sun et al., 2011) and so are not shown for clarity.

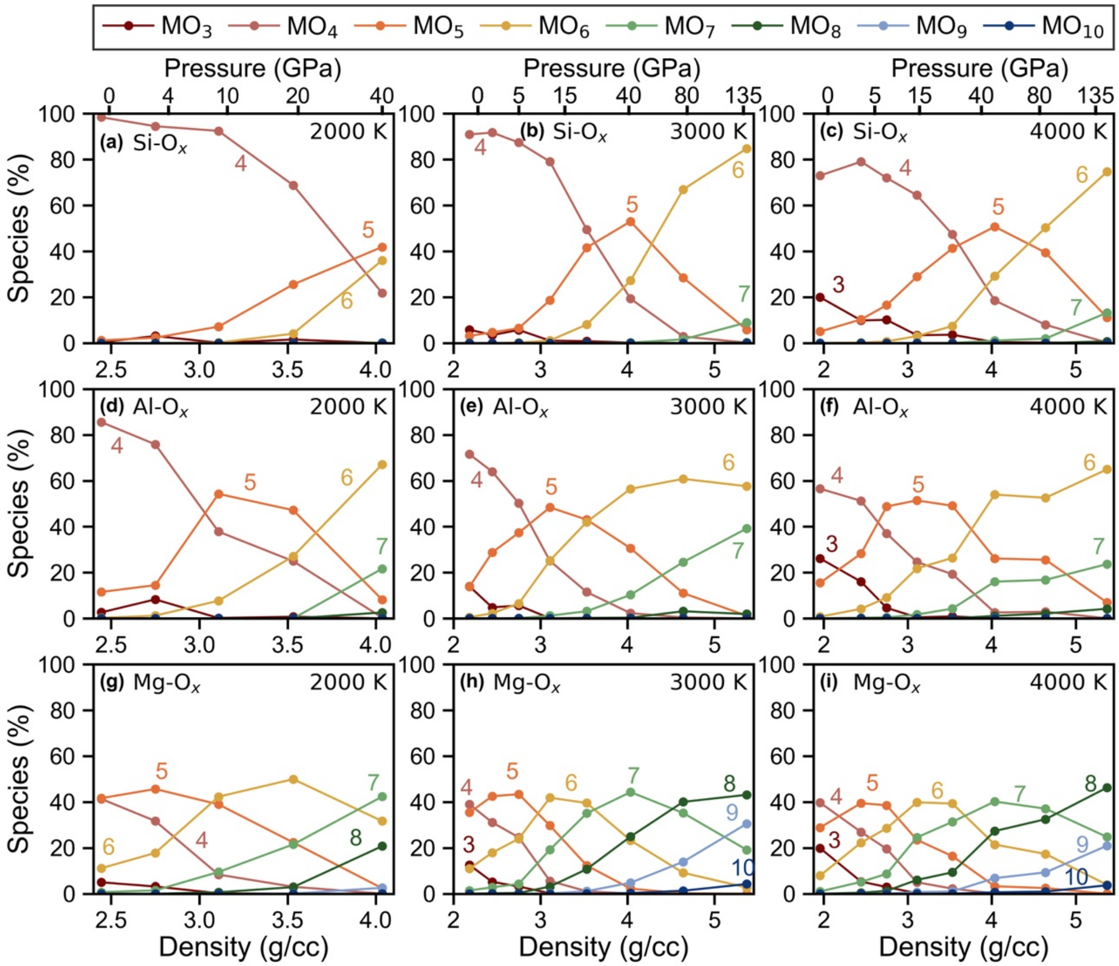

Figure S4. Atomic coordination of silicon at 2000 K (a), silicon at 3000 K (b), silicon at 4000 K (c), aluminum at 2000 K (d), aluminum at 3000 K (e), aluminum at 4000 K (f), magnesium at 2000 K (g), magnesium at 3000 K (h), and magnesium at 4000 K (i) with respect to oxygen as a function of density (corresponding pressures are provided on top). Coordination numbers are labeled next to the corresponding curves.

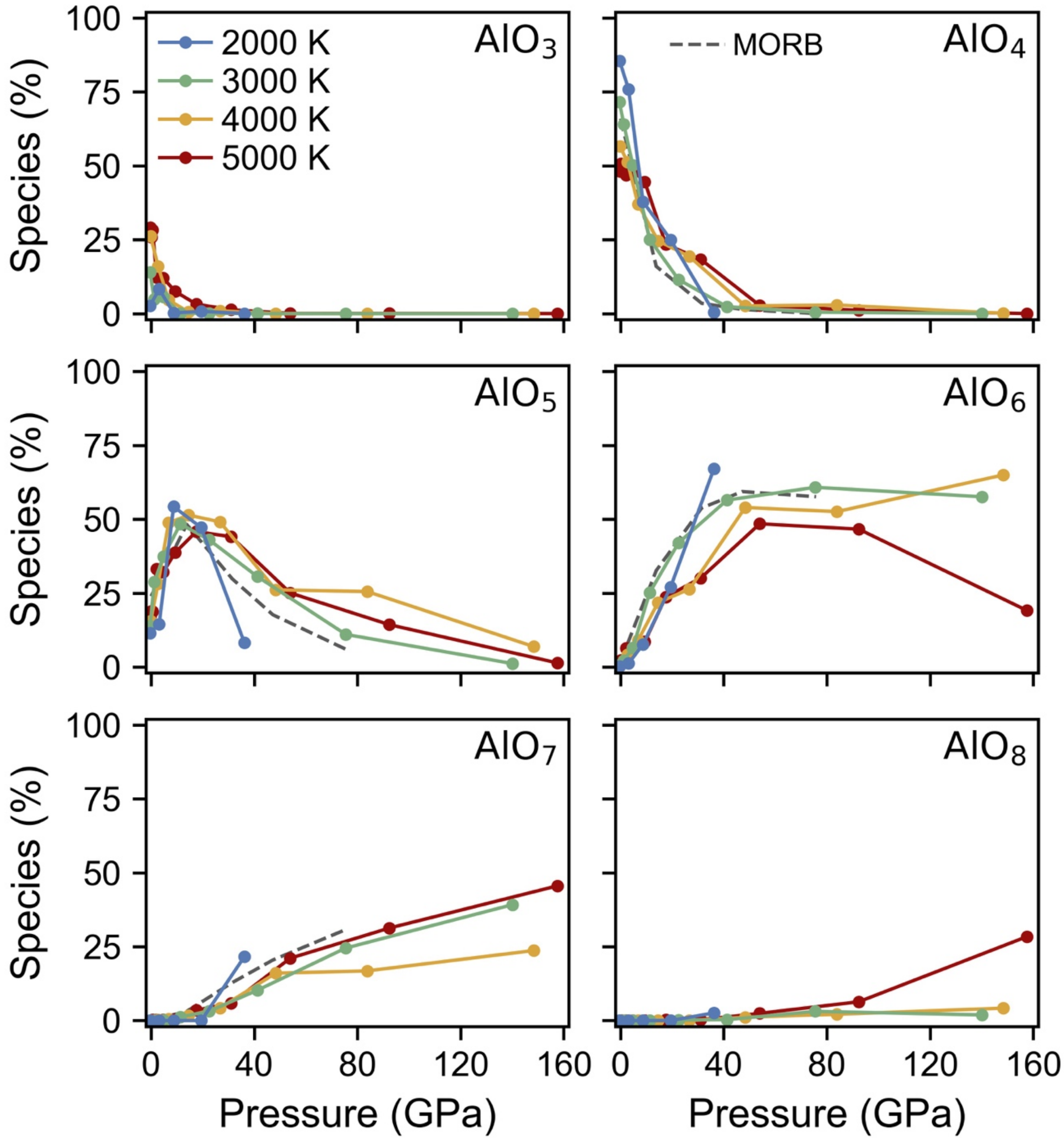

Figure S5. The proportions of the six most common species of AlO_x as a function of pressure. The proportions of AlO_x species from ab initio molecular dynamics calculations on MORB melt are shown as the dashed gray curve, averaged over temperatures of 1800-4000 K and pressures at each volume (Bajgain et al., 2015).

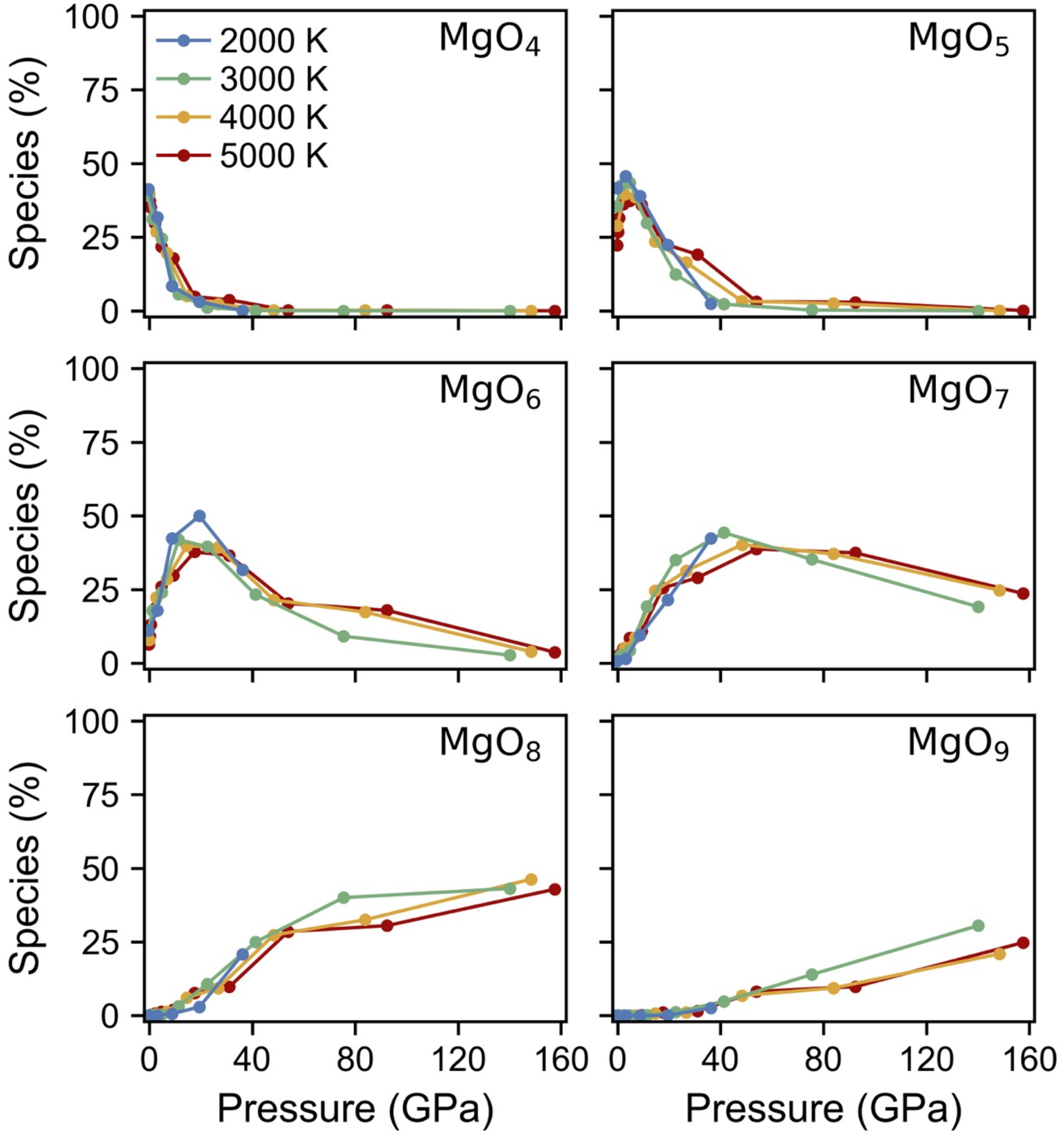

Figure S6. The proportions of the six most common species of MgO_x as a function of pressure.

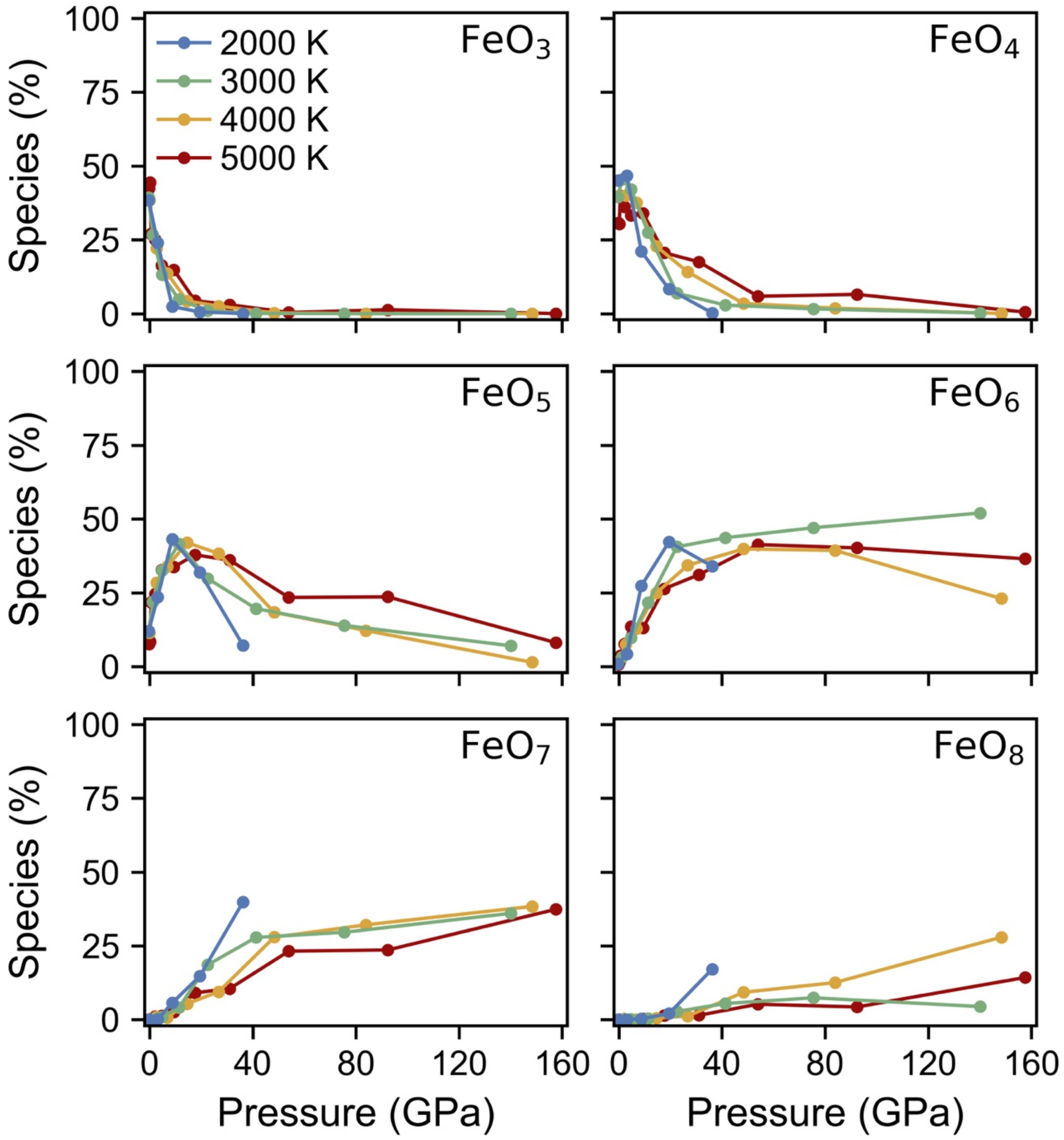

Figure S7. The proportions of the six most common species of FeO_x as a function of pressure.

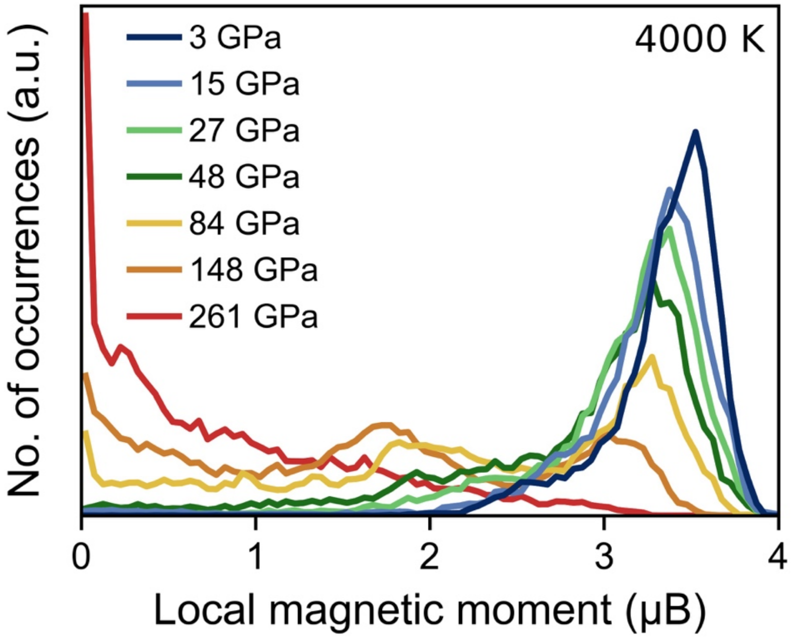

Figure S8. The distribution of local magnetic moments in iron, as computed inside the PAW spheres, as a function of pressure at a temperature of 4000 K. At ambient pressure, iron has an average magnetic moment of 3.5 μB, decreasing to 2.8 μB by 48 GPa and 2.2 μB by 84 GPa.

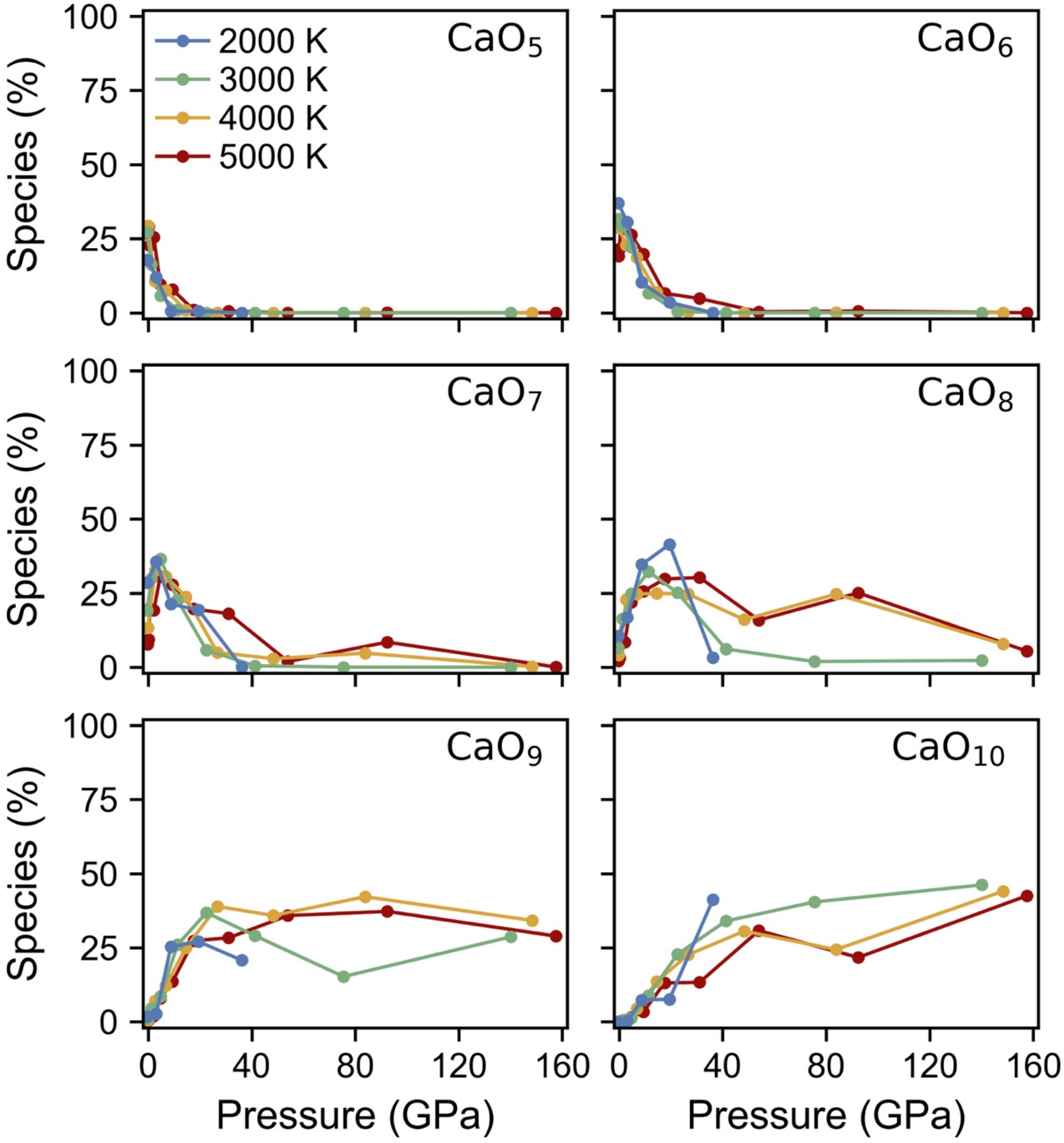

Figure S9. The proportions of the six most common species of CaO_x as a function of pressure.

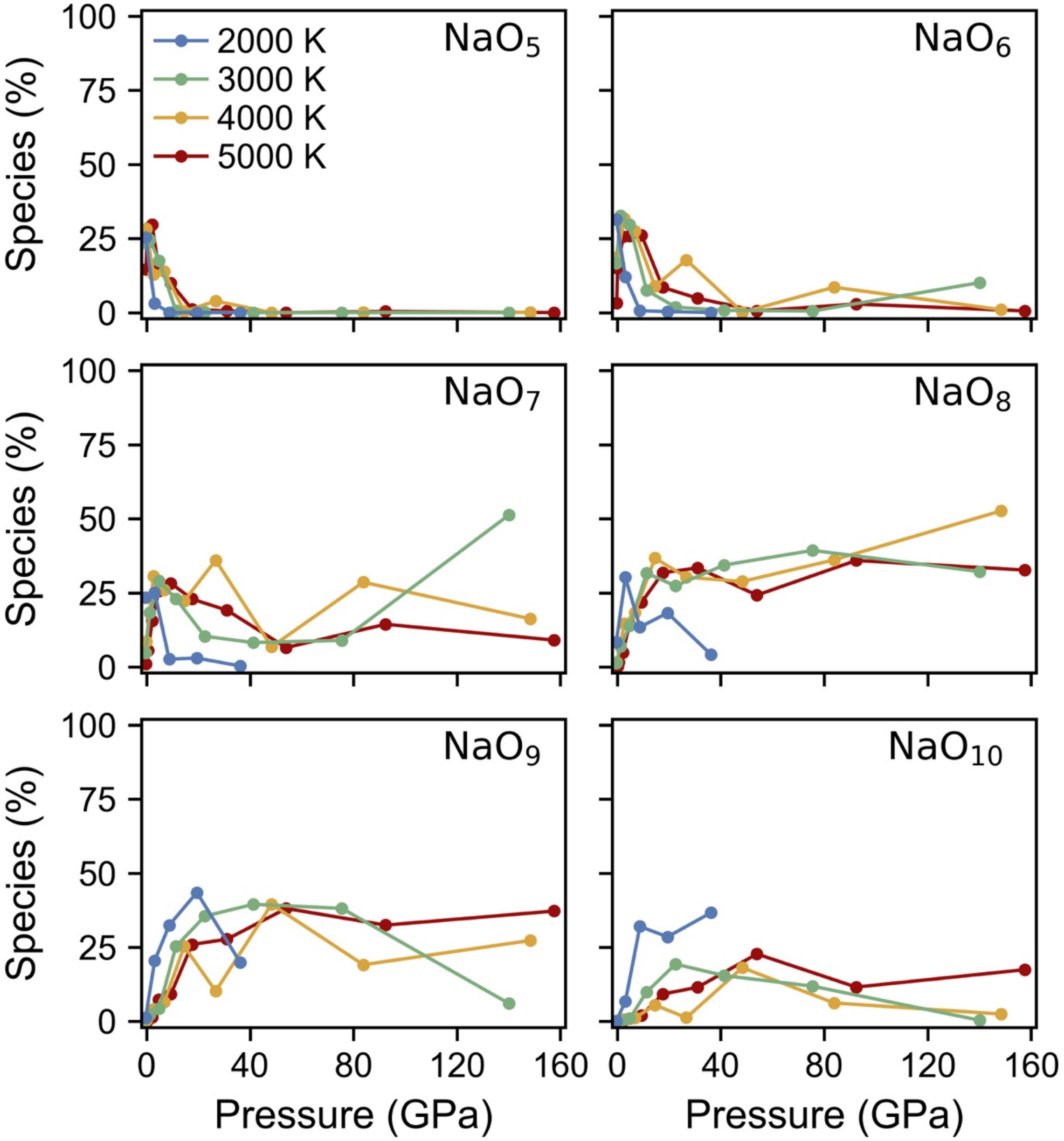

Figure S10. The proportions of the six most common species of NaO_x as a function of pressure.

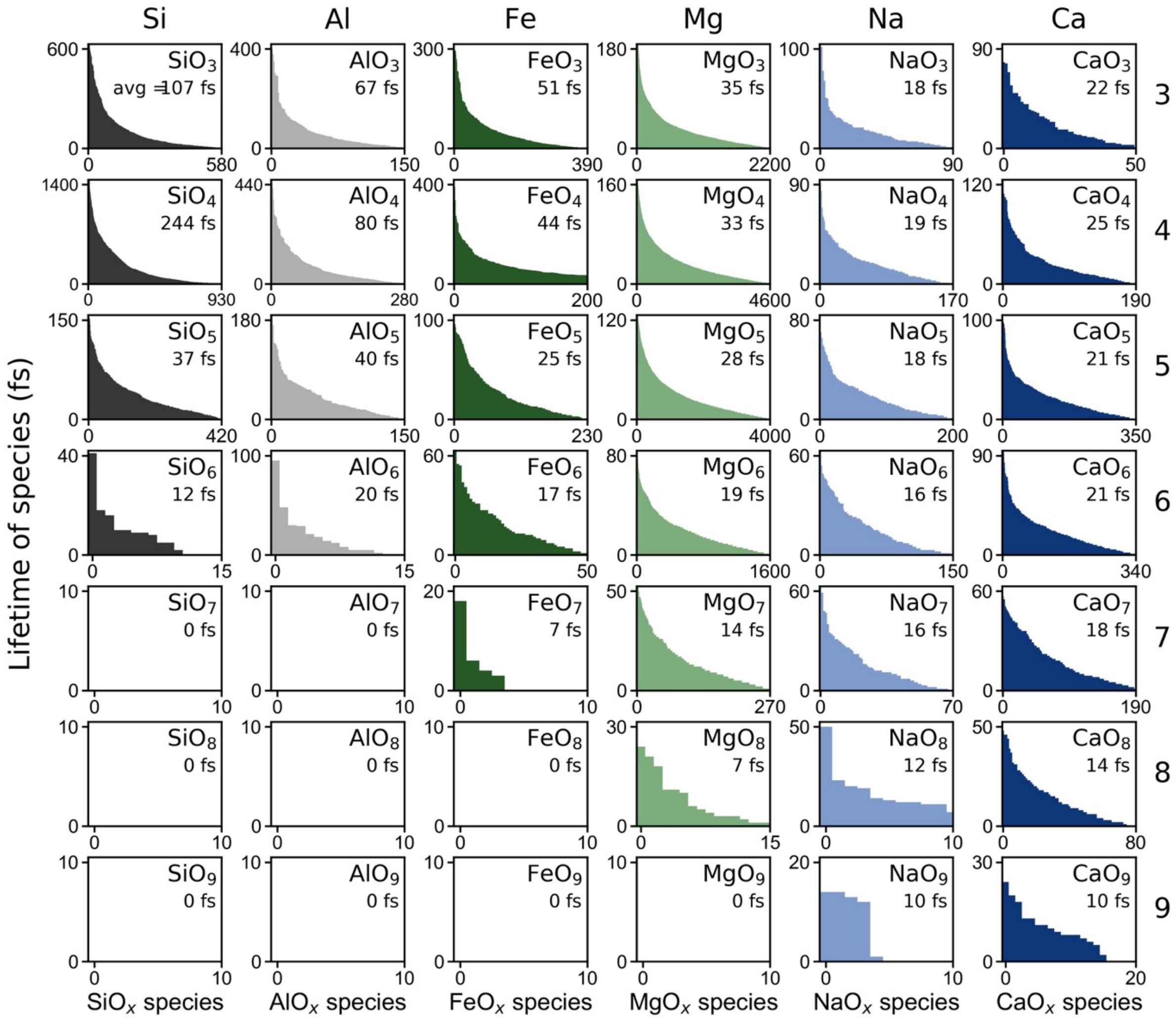

Figure S11. Lifetimes of each species at 0 GPa and 4000 K. The x-axis represents each individual MO_x species sorted in decreasing order of lifetimes and the y-axis is the lifetime (the amount of time each species lived for) in femtoseconds. The average length of time that each type of species existed for in femtoseconds is labeled within each figure. The total simulation length was 13 picoseconds.

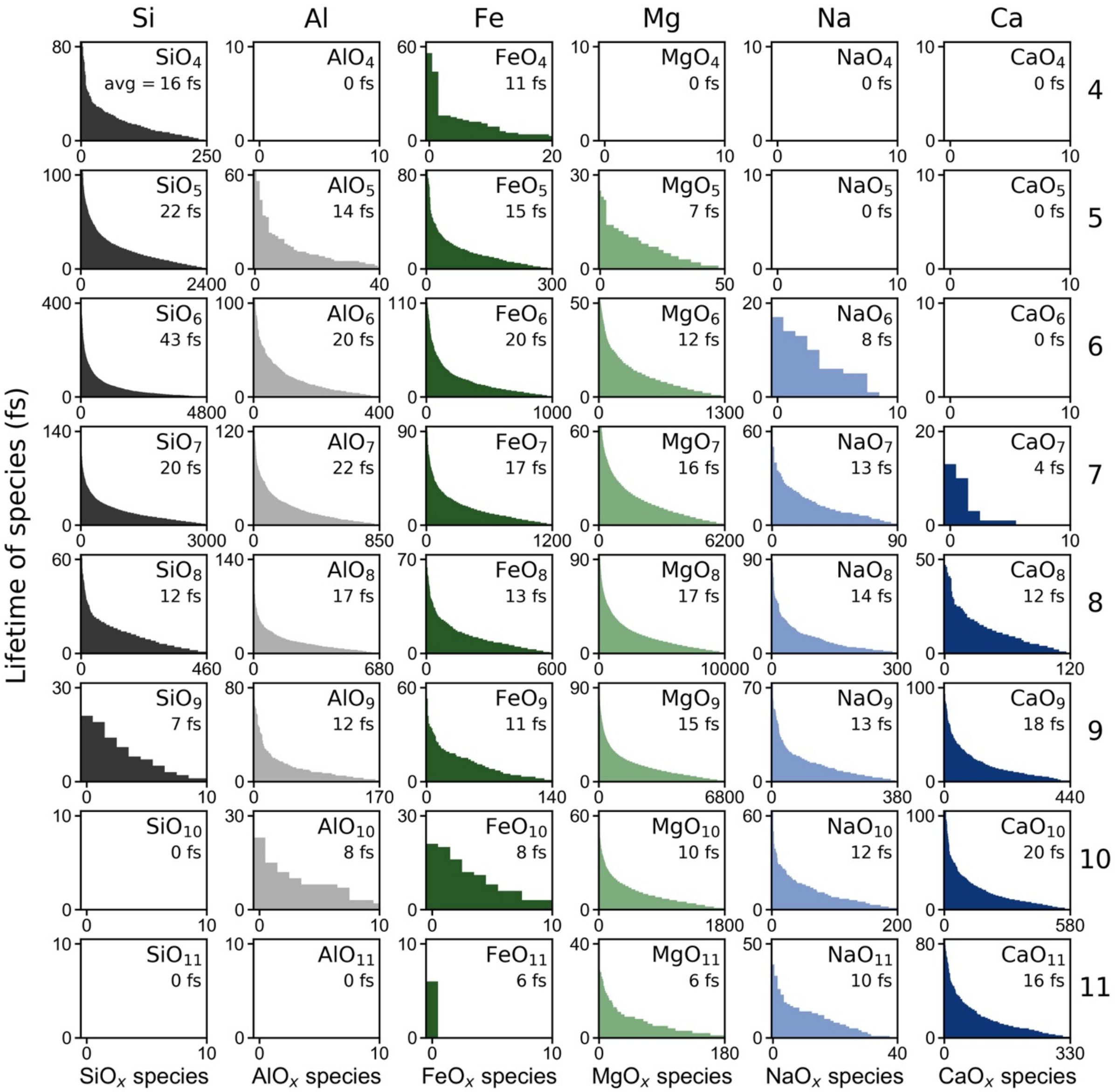

Figure S12. Lifetimes of each species at 150 GPa and 5000 K. The x-axis represents each individual MO_x species sorted in decreasing order of lifetimes and the y-axis is the lifetime (the amount of time each species lived for) in femtoseconds. The average length of time that each type of species existed for in femtoseconds is labeled within each figure. The total simulation length was 14 picoseconds.

Pressure (GPa)	Si-O	Al-O	Mg-O	Fe-O	Ca-O	Na-O
-0.4±1.1	4.0	4.1	4.6	3.7	6.3	6.0
3.1±1.3	4.0	4.1	4.8	4.1	6.6	7.8
8.7±1.6	4.1	4.7	5.5	5.1	8.0	9.5
19.4±2.6	4.3	5.0	6.0	5.7	8.2	9.2
36.2±1.9	5.1	6.2	6.9	6.7	10.1	10.2

Table S1. Average coordination of cations with respect to oxygen at 2000 K. Pressure errors are one standard deviation, as calculated from the simulations.

Pressure (GPa)	Si-O	Al-O	Mg-O	Fe-O	Ca-O	Na-O
-0.4±1.3	4.0	4.0	4.5	3.6	5.8	4.5
1.2±1.4	4.0	4.3	4.8	4.0	6.6	5.8
4.7±1.5	4.0	4.4	5.0	4.4	7.1	6.5
11.3±1.6	4.2	5.0	5.8	4.9	8.1	8.1
22.5±1.9	4.6	5.4	6.5	5.8	9.0	8.8
41.3±4.2	5.1	5.8	7.1	6.1	9.9	8.7
75.5±2.8	5.7	6.2	7.6	6.3	10.4	8.6
140.1±2.6	6.0	6.4	8.1	6.4	9.9	7.4

Table S2. Average coordination of cations with respect to oxygen at 3000 K. Pressure errors are one standard deviation, as calculated from the simulations.

Pressure (GPa)	Si-O	Al-O	Mg-O	Fe-O	Ca-O	Na-O
-0.2±1.3	3.8	3.9	4.2	3.6	5.4	4.8
2.7±1.5	4.0	4.2	5.0	4.2	6.9	6.5
6.8±1.7	4.1	4.6	5.2	4.5	7.3	6.6
14.5±1.9	4.3	5.0	6.0	5.1	8.3	8.0
26.7±2.2	4.5	5.1	6.3	5.4	9.1	7.3
48.3±2.2	5.1	5.9	7.1	6.2	9.4	8.9
83.8±2.7	5.5	5.9	7.3	6.5	9.0	7.9
148.3±2.9	6.0	6.3	8.0	7.2	9.6	8.1

Table S3. Average coordination of cations with respect to oxygen at 4000 K. Pressure errors are one standard deviation, as calculated from the simulations.

Pressure (GPa)	Si-O	Al-O	Mg-O	Fe-O	Ca-O	Na-O
-0.3±1.2	3.5	3.8	4.0	3.3	4.8	3.4
0.1±1.2	3.5	3.9	4.2	3.4	5.0	4.3
0.6±1.4	3.7	3.9	4.5	3.9	5.8	4.7
2.1±1.6	3.8	4.3	4.8	4.1	5.9	5.5
4.6±1.6	4.0	4.3	5.2	4.4	6.9	6.5
9.4±1.8	4.0	4.5	5.3	4.5	7.3	6.9
17.5±2.1	4.4	5.0	6.1	5.2	8.3	8.0
31.1±2.3	4.6	5.2	6.3	5.3	8.4	8.3
54.0±2.7	5.1	6.0	7.2	6.0	9.4	9.0
92.4±3.0	5.4	6.3	7.3	5.9	8.9	8.4
157.6±3.2	6.0	7.2	8.0	6.7	9.9	8.7

Table S4. Average coordination of cations with respect to oxygen at 5000 K. Pressure errors are one standard deviation, as calculated from the simulations.
